# Interactions of Salicyluric Acid with Metal Ions and Ascorbic Acid: Possible Biological and Toxicity Implications in Aspirin Use

**DOI:** 10.3390/biology15191740

**Published:** 2026-10-01

**Authors:** Viktor A. Timoshnikov, Aleksandr A. Deriskiba, Vladimir E. Koshman, Nikolay E. Polyakov, George J. Kontoghiorghes

**Affiliations:** 1 Institute of Chemical Kinetics & Combustion, 630090 Novosibirsk, Russia; timoshnikov@kinetics.nsc.ru (V.A.T.); a.deriskiba@g.nsu.ru (A.A.D.); kosmanvova2010@mail.ru (V.E.K.); 2 Department of Physics, Novosibirsk State University, 630090 Novosibirsk, Russia; 3 Postgraduate Research Institute of Science, Technology, Environment and Medicine, CY-3021 Limassol, Cyprus

**Keywords:** salicyluric acid, aspirin metabolite, chelation, deferiprone, ascorbic acid, competition, complex stability, iron, copper, aluminum

## Abstract

**Simple Summary:**

Salicyluric acid (SUA) is a natural plant product and the major metabolite of the widely used drug aspirin. Iron-deficiency anemia has been identified as one of the toxic side effects of long-term aspirin use, suggesting the possible implication of SUA and other chelating metabolites as the cause. The biological, toxicological, and clinical effects of SUA, including interactions with essential and xenobiotic metals, have not yet been fully investigated. In this study, SUA formed moderately stable complexes with iron, copper, and aluminum ions, which under certain conditions can promote oxidative damage to lipid molecules. However, in the presence of vitamin C, this oxidative effect was modulated by the specific type of transition metal, highlighting a complex interaction between these components. The results of these interactions may contribute to a better understanding of the biological effects of SUA and the management of aspirin’s side effects, as well as the design of improved therapeutic strategies.

**Abstract:**

Aspirin is one of the most widely used pharmaceuticals, yet its long-term use is associated with some side effects, including iron-deficiency anemia, which has been mainly linked to the iron-chelating properties of its major metabolite, salicyluric acid (SUA). In this study, the chelating and redox properties of SUA in the presence of iron (Fe^3+^), copper (Cu^2+^), and aluminum (Al^3+^), under neutral pH conditions were investigated using UV-Vis spectrophotometry, Job plot analysis, and ^1^H NMR spectroscopy. The stability constants determined for SUA complexes with Cu^2+^ and Al^3+^ were K = 9.9 × 10^6^ and 1.40 × 10^4^ M^−1^, respectively, classifying SUA as a moderate-affinity chelator. No detectable complexes of SUA with Fe^2+^ or Cu^+^ were observed, indicating selectivity of SUA for higher oxidation states. Competition experiments with the chelating drug deferiprone (L1) confirmed the quantitative displacement of SUA from metal coordination spheres. In a Fenton-initiated lipid peroxidation model using linoleic acid micelles, SUA–copper and iron metal complexes accelerated lipid oxidation, demonstrating pro-oxidant activity. However, the combination of SUA and vitamin C resulted in a net decrease in the lipid peroxidation rate compared to the system containing SUA alone, suggesting a complex interplay between chelation and redox modulation.

## 1. Introduction

Acetylsalicylic acid (aspirin) is one of the most widely recognized drugs in modern pharmacology and has been actively used for over a century to treat a broad spectrum of conditions [1]. This drug exhibits antiplatelet, antipyretic, and anti-inflammatory properties and is employed in both the treatment and prevention of various ailments, including cardiovascular diseases, colds, influenza, migraines, and others [2,3,4]. Over the 20th and 21st centuries, numerous studies have investigated its pharmacokinetics, molecular mechanisms of action, and its anticancer, neuroprotective, and other properties [2,3,4].

**Figure 1:**
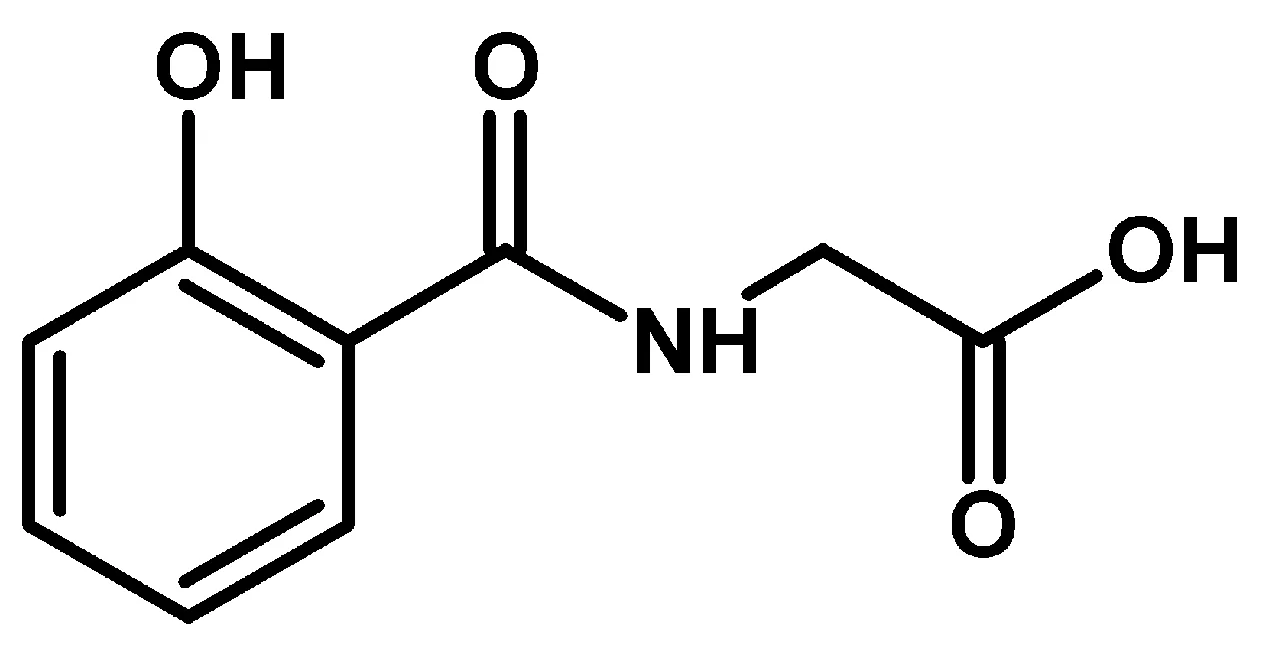
The molecular structure of salicyluric acid (SUA).

Despite the effectiveness of aspirin and its applicability for the treatment of many diseases, this drug has a number of side effects, especially with long-term use. The main ones are internal bleeding caused by a decrease in platelet aggregation and damage to the gastrointestinal mucosa [5,6]. In addition, negative effects are caused not only by aspirin itself, but also by its metabolic products. Among such negative consequences, one can highlight the disruption of ATP production due to uncoupling of oxidative phosphorylation [7]. In addition, a correlation has been established between long-term use of aspirin and the development of iron-deficiency anemia not associated with bleeding in a significant proportion of users [8,9,10]. It has been hypothesized that this relationship may be due to the ability of aspirin metabolites to chelate iron and promote its excretion from the body [8,9]. Among the primary metabolites, salicyluric acid (SUA) remains the least studied (Figure 1).

The structure of SUA contains carboxyl, hydroxyl, and amide groups potentially capable of coordinating metal ions (Figure 1). Several studies have examined the chelation properties of SUA with iron and other metal ions in acidic media [11,12,13]. However, the chelating properties of SUA in neutral media have not been fully investigated. To systematically evaluate these properties, it is essential to focus on metal ions that are critically involved in biological homeostasis, oxidative stress, or specific drug–drug interactions. In this study, iron (Fe^3+^/Fe^2+^) and copper (Cu^2+^/Cu^+^) were chosen as key endogenous transition metals due to their prominent roles in cell metabolism and their dangerous propensity to catalyze reactive oxygen species (ROS) generation when left unchelated. Additionally, aluminum (Al^3+^), which possesses a high charge-to-size ratio similar to Fe^3+^ and frequently interferes with mineral pathways, was selected as a representative toxic xenobiotic metal. Preliminary studies have suggested that SUA can be classified as a moderately active chelator, demonstrating the maximum stability constant for Fe^3+^ at low pH (max log K = 15 at pH 2.25), which decreases with increasing pH [13]. Such low stability constants do not play a significant role in metal ion homeostasis during short-term administration of the drug. However, under long-term use, they may potentially affect metabolic pathways and interfere with the labile pools of iron and other metal ions.

Questions also arise regarding SUA competing with other endogenous and exogenous chelators. Among these, ascorbic acid (Asc) deserves particular attention, since it is widely available in foods such as fruit and vegetables and also sold as a nutraceutical. Ascorbic acid is one of the best-known antioxidants and used in the treatment of a wide range of diseases, including scurvy, pneumonia, type 2 diabetes, cancer and Alzheimer’s disease, among many others, as a primary drug or in adjuvant therapy [14,15,16,17,18]. However, Asc can also reduce the oxidized forms of copper and iron ions, converting them into a more reactive state, as described below (1) and (2) [14,19]:

(1) $${Fe}^{3+}+Asc\to {Fe}^{2+}+{\dot{Asc}}^{+}$$

(2) $${Cu}^{2+}+Asc\to {Cu}^{+}+{\dot{Asc}}^{+}$$

In this context, the reduction of transition metal ions can lead to enhanced oxidative stress associated with the generation of reactive oxygen species (ROS) through the participation of reduced ions in redox reactions, such as the Fenton reaction, as described below (3)–(6) [20,21,22,23]. Conversely, for copper, the oxidized Cu^2+^ state is often more pro-oxidant and reactive in driving lipid peroxidation than its reduced Cu^+^ counterpart [24].

(3) $${Fe}^{2+}+H_{2}O_{2}\to {Fe}^{3+}+\dot{OH}+{OH}^{-}$$

(4) $${Fe}^{3+}+H_{2}O_{2}\to {Fe}^{2+}+\dot{OOH}+H^{+}$$

(5) $${Cu}^{+}+H_{2}O_{2}\to {Cu}^{2+}+{OH}^{-}+\dot{OH}$$

(6) $${Cu}^{2+}+H_{2}O_{2}\to {Cu}^{+}+\dot{OOH}+H^{+}$$

This pro-oxidant risk highlights the dual role of iron valence states in biology. On the one hand, from a therapeutic standpoint, ascorbic acid is widely used to treat iron-deficiency anemia by converting poorly absorbed Fe^3+^ into the more readily absorbable Fe^2+^ within the gastrointestinal tract [14,25]. Therefore, Asc is often co-administered with iron-containing medications [26]. On the other hand, once absorbed, free Fe^2+^ ions can stimulate the aforementioned harmful ROS production. This dualism raises the question of how Asc may affect the stability and crucially the redox activity of SUA chelate complexes with transition metal ions. Essentially, understanding whether SUA can safely bind and inactivate this highly reactive Fe^2+^ is vital for assessing its overall impact on oxidative stress. It should be noted that Asc itself can coordinate Fe^2+^ in solution [14,27].

Given that SUA acts as an unintended chelator, its behavior is best understood by comparison with clinical chelating agents. Although targeted iron chelators like deferiprone (L1) are classified as orphan drugs used exclusively in highly specific, rare clinical cases of transfusional iron overload, L1 serves as an excellent reference model for iron-binding studies due to its well-characterized coordination chemistry. Deferiprone, or 1,2-dimethyl-3-hydroxypyrid-4-one, is a drug used to treat iron overload disorders [28]. This compound has relatively high stability constants for complexes with both iron (log K_L1-Fe(III)_ = 36.7, per three ligands) and copper (log K_L1-Cu(II)_ = 19.9, per two ligands) [29]. However, although the expected stability constants of SUA chelate complexes with metal ions are several orders of magnitude lower than those of L1 under physiological conditions, the behavior of these chelate complexes remains an open question due to the possible incorporation of multiple different chelates into the inner coordination sphere, as described in other studies [30].

In addition to essential transition metal ions, interactions with xenobiotic metal ions are also of interest, including metal ions such as aluminum, which is found in water, food products, and many pharmaceuticals. For example, excessive use of aluminum-based antacid drugs can lead to adverse effects, ranging from gastrointestinal dysfunction to mineral metabolism disorders and even encephalopathy, following prolonged use [31,32,33]. It should be noted that cases of the combined use of aspirin and antacids have also been documented in the literature. Specifically, combined use reduces the efficacy of aspirin absorption, resulting in decreased plasma concentrations of aspirin and increased urinary excretion (altered renal clearance), probably due to its reduced bioavailability during absorption [34,35]. It has also been reported that long-term co-administration of these drugs can exacerbate iron-deficiency anemia [8].

This study aimed to investigate the chelating properties of the aspirin metabolite SUA toward the essential transition metal ions iron and copper, and also the xenobiotic metal ion aluminum. Furthermore, the influence of the chelators L1 and Asc on the stability and redox activity of SUA chelate complexes with iron, copper, and aluminum was also examined. To address these objectives, UV-Vis spectrophotometry and NMR spectroscopy in conjunction with an experimental model of linoleic acid micelle peroxidation was used. The findings of these studies will enhance the current understanding of the chelating activity of SUA, the main aspirin metabolite, its stability, and its redox activity in the presence of other natural or synthetic chelating agents. Furthermore, these SUA interactions may also show possible biological effects in relation to iron and copper metabolism and toxicity and also other effects related to aluminum toxicity.

## 2. Materials and Methods

### 2.1. Materials

Ferrous sulfate (FeSO_4_·7H_2_O, 99%), ferric nitrate (Fe(NO_3_)_3_·6H_2_O), copper chloride (CuCl_2_·2H_2_O, 99%), aluminum chloride (AlCl_3_, 99%), ascorbic acid (Asc, >95%), sodium 1,4-piperazinediethanesulfonate (Na-PIPES, ≥99%), and N-(2-hydroxyethyl)piperazine-N′-(2-ethanesulfonic acid) (HEPES, ≥99.5%) were obtained from Sigma-Aldrich (St. Louis, MO, USA). Deferiprone (L1) was obtained from LIPOMED Inc., Arlesheim, Switzerland. Linoleic acid (LA, >99.0%) and salicyluric acid (2-hydroxyhippuric acid, SUA, 95%) were purchased from Shanghai Aladdin Bio-Chem Technology Co., Ltd. (Shanghai, China). All compounds were used as received. Deuterium oxide (D_2_O, 99.8% D) was obtained from Solvex-D Co. (Moscow, Russia) and used as supplied.

### 2.2. Methods

#### 2.2.1. UV-Vis Metal Coordination Study

UV-Vis optical absorbance spectra and kinetics were measured in aqueous buffer solutions using polystyrene cuvettes on an SF-2000 spectrophotometer (OKB SPECTR LLC, Saint Petersburg, Russia) over the 400–1000 nm range and using quartz cuvettes over the 300–900 nm range. The optical path length of the cuvettes was 1 cm. HEPES (0.1 M), in which all stock solutions were pre-dissolved, served as the buffer for all UV-Vis experiments. After mixing the components and during titration, the pH of the final solutions was carefully adjusted to 7.0 with KOH to prevent precipitation of insoluble metal hydroxides.

The continuous variation method (Job plot) was used to study the stoichiometry of the SUA chelate complexes with Cu^2+^, Fe^3+^, and Al^3+^ ions [36]. For a series of samples with a constant total concentration of metal and chelator, the mole fraction of the chelator was varied from 0 to 1. The UV-Vis absorbance was measured at a wavelength in the absorption region of the chelate complex. The stoichiometry of the complex was determined from the position of the maximum on the plot of absorbance versus chelator mole fraction. In this context, if a single stoichiometry predominates in solution, the resulting curve will exhibit a dome like shape with a clearly defined maximum corresponding to the metal-to-chelator stoichiometry. For Cu^2+^, the total concentration of SUA and CuCl_2_ was 5 mM, for Fe^3+^ 1 mM, and for Al^3+^ 0.5 mM. After mixing, all solutions were allowed to stand for 30 min to reach thermodynamic equilibrium.

To determine the stability constants and molar extinction coefficients of the metal complexes, the solutions were titrated at a constant SUA concentration with a varying metal salt concentration. For experiments with Fe^3+^, the metal salt concentration was fixed and the SUA concentration was varied. The absorbance at the characteristic wavelength of the complex was then plotted as a function of the metal-to-chelator ratio (or chelator-to-metal ratio for Fe^3+^). A general equation describing the absorbance of the solution during complex formation was used to fit the resulting data, as follows:

(7) $$D=\sum_{n,m}\varepsilon_{nm}\left[M_{n}L_{m}\right]+\varepsilon_{L}\left[L\right]+\varepsilon_{M}\left[M\right]$$

where ε_nm_ is the molar extinction coefficient of the complex with the corresponding stoichiometry and n and m are the stoichiometric coefficients for each metal, as determined from the Job plot.

The concentration of the complex M_n_L_m_ can be generally expressed as a function of the total concentrations of the components and the stability constant K, as follows:

(8) $$K=\frac{\left[M_{n}L_{m}\right]}{{\left[M\right]}^{n}\times {\left[L\right]}^{m}}$$

where [M] and [L] are the equilibrium concentrations of free metal and free chelator, respectively, as calculated from the mass balance equations:

(9) $${\left[M\right]}_{0}=[M]+\sum_{n,m}n\left[M_{n}L_{m}\right]$$

(10) $${\left[L\right]}_{0}=[L]+\sum_{n,m}m\left[M_{n}L_{m}\right]$$

The system of nonlinear equations was numerically solved by global nonlinear regression analysis using the SciPy package for Python (ver. 3.10.4), which was used to develop the calculation program. The parameters K and ε were varied until the sum of squared deviations between the experimental and calculated absorbance values was minimized.

#### 2.2.2. ^1^H NMR Study of Linoleic Acid Peroxidation

^1^H NMR measurements were performed using a Bruker Avance HD III NMR spectrometer (operating at 500 MHz, Bruker BioSpin GmbH, Rheinstetten, Germany). The 90° and 180° pulse lengths were 10.9 μs and 21.8 μs, respectively. All NMR spectra were processed using TopSpin software (ver. 4.0.5). All experiments were conducted at 303 K.

To study the effect of SUA and Asc on the peroxidation of LA micelles, samples were prepared as follows. LA was dissolved in chloroform and then dried under nitrogen to minimize autoxidation. The lipid film was then hydrated with 10 mM Na-PIPES buffer and sonicated for 1 h to obtain a micellar solution. For each metal ion (Cu^2+^ and Fe^3+^), the following peroxidation experiments were conducted: LA + metal; LA + metal + SUA; LA + metal + Asc; LA + metal + SUA + Asc. In each experimental series, prior to adding the next component (except for experiments with Asc), the solutions were incubated for 15 min to reach thermodynamic equilibrium. The peroxidation reaction was initiated by the addition of H_2_O_2_. In experiments with Asc, the reaction was initiated by simultaneous addition of Asc and H_2_O_2_ to the solution. Final concentrations were LA 3.5 mM, metal salts (CuCl_2_ and Fe(NO_3_)_3_) 0.1 mM each, SUA 1 mM, Asc 0.5 mM, and H_2_O_2_ 0.5 M.

Sodium PIPES (Na-PIPES, 10 mM) was used as the buffer for the ^1^H NMR experiments. It should be noted that in contrast to HEPES, which was used in the UV-Vis experiments, the use of Na-PIPES does not produce signals in the region around 2.7 ppm. This region contains the signal from the bis-allylic proton of LA, the integrated intensity of which reflects its concentration in solution.

#### 2.2.3. Data Fitting and Statistical Analysis

To determine the definitive values of the stability and extinction constants of chelate complexes, as well as the effective constants of linoleic acid degradation in the peroxidation reaction, experimental datasets obtained from three independent replicates were analyzed simultaneously using a global fitting approach. Global nonlinear regression was performed using Python 3 software.

## 3. Results and Discussion

### 3.1. Coordination Studies of SUA with Fe^3+^, Cu^2+^ and Al^3+^ in Water at pH 7

The first stage of this work was to study the formation of SUA chelate complexes with metal ions in neutral media. Although chelate complexes in acidic media have been described elsewhere [11,12], data on their stability in neutral media are virtually nonexistent. A key consideration in experiments designed to study the coordination activity of moderate-affinity chelators is the selection of a buffer that does not coordinate metal ions and does not absorb light in the measurement range. For spectrophotometric experiments, the Good buffer HEPES was used [37]. All stock solutions were prepared in 0.1 M buffer (pH 5.2), and then the pH was carefully adjusted to 7.0 to prevent precipitation of insoluble metal hydroxides [38,39].

**Figure 2:**
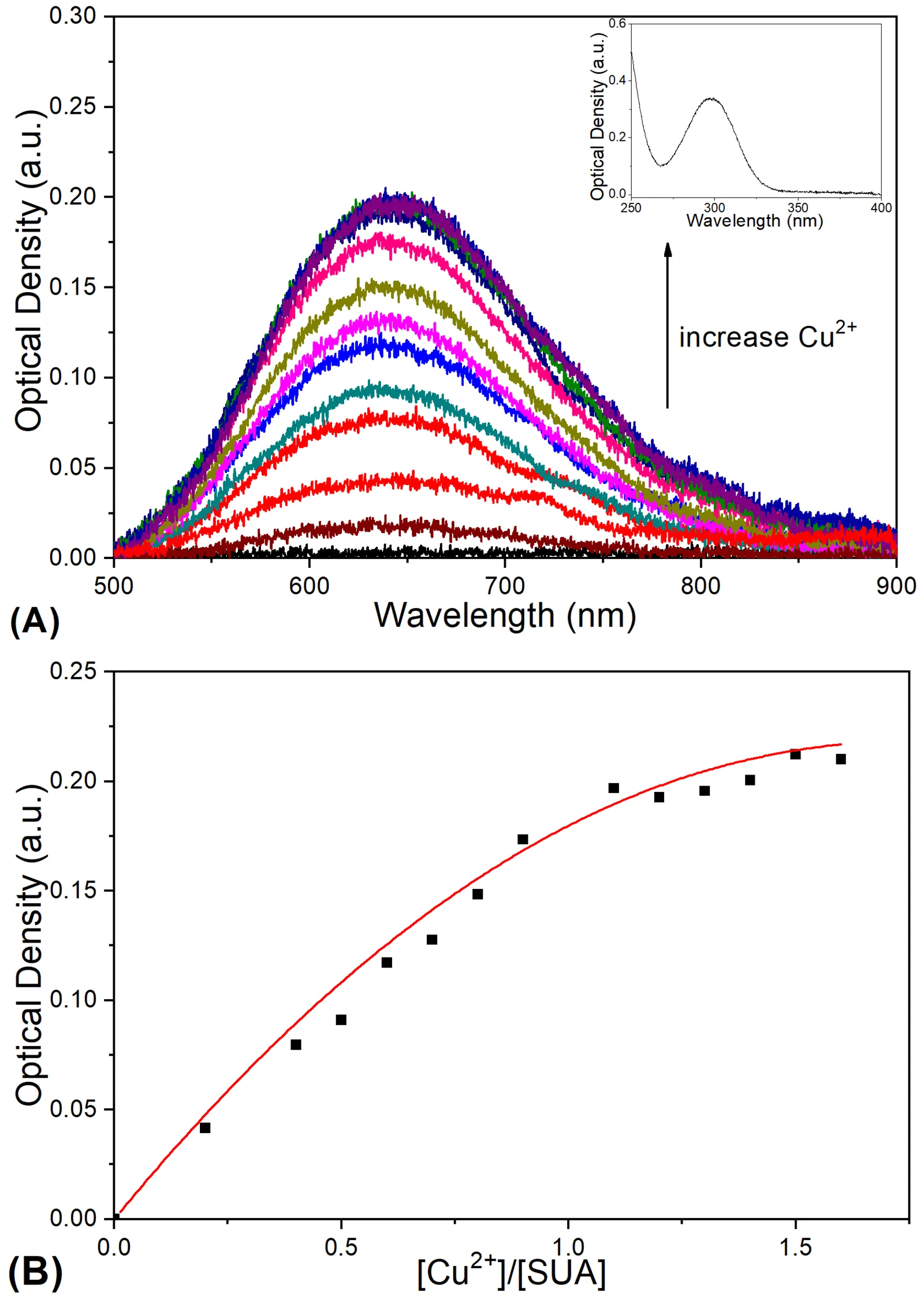

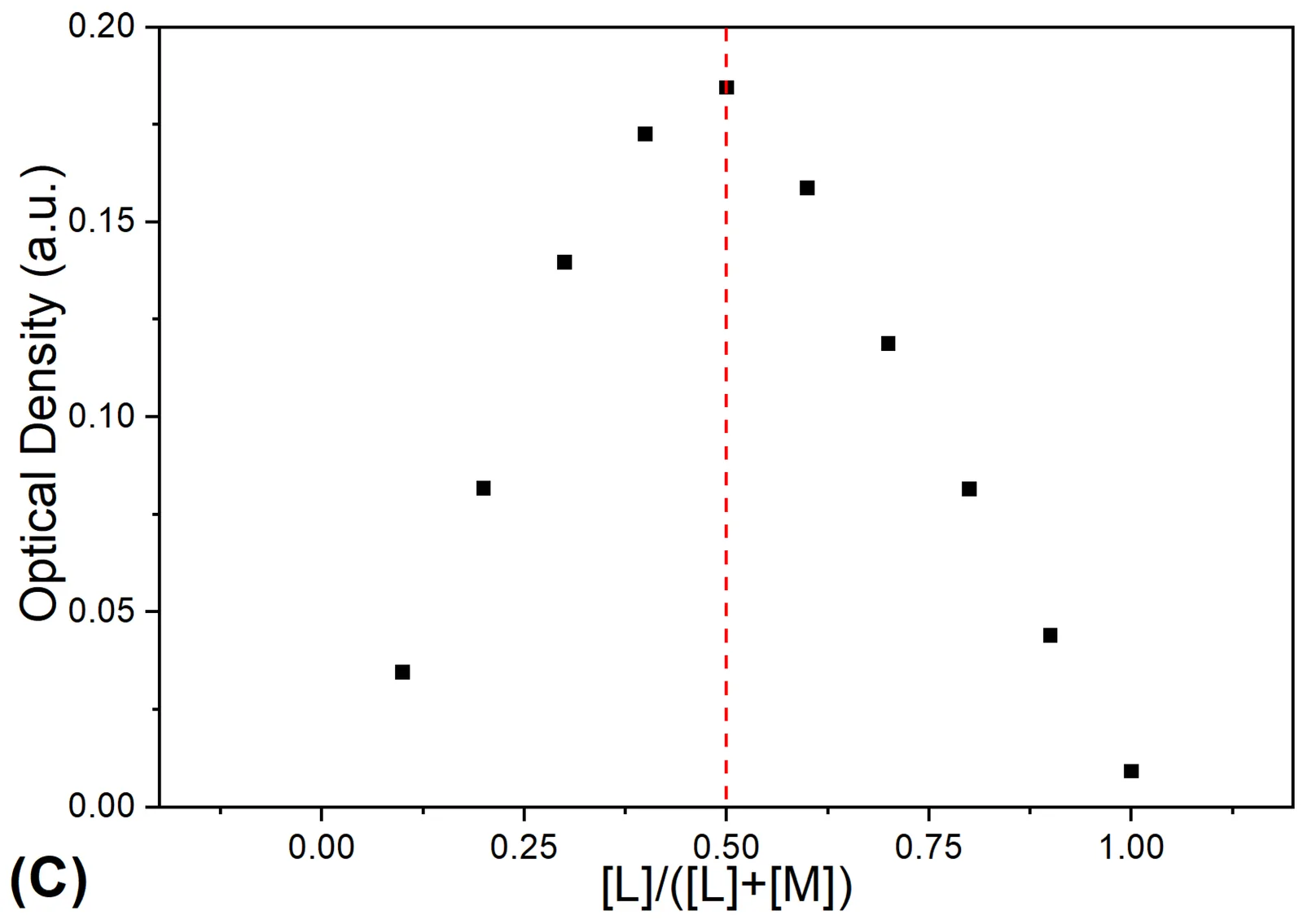
(**A**) UV-Vis absorbance spectra of CuCl_2_ solutions (0–2.75 mM) in the presence of 2.5 mM SUA. Inset: UV-Vis spectrum of 0.1 mM SUA in buffer. (**B**) Absorbance of CuCl_2_ solutions (0–2.75 mM) in the presence of 2.5 mM SUA at 650 nm as a function of the [Cu^2+^]/[SUA] ratio. The solid red curve represents the fit obtained by solving Equations (11)–(14). (**C**) Job plot (continuous variation diagram) for SUA–Cu^2+^ mixtures at 650 nm. Total concentration of SUA and Cu^2+^ was 5 mM. The red dashed line indicates the maximum of the Job plot. All experiments were conducted in 0.1 M HEPES buffer (pH 7.0, adjusted with KOH) at 25 °C.

To determine the stability constant of the SUA complex with Cu^2+^, the UV-Vis spectra of the SUA solutions at various Cu^2+^ concentrations were first recorded (Figure 2A). Upon addition of the copper salt, an absorption band appeared in the 500–800 nm region, characteristic of the SUA–Cu^2+^ chelate complex.

From the data obtained in Figure 2A, the absorbance at the characteristic wavelength of the complex (650 nm) was then plotted as a function of the [Cu^2+^]/[SUA] ratio (Figure 2B). Under these conditions, the curve reached a plateau after the 1:1 ratio, indicating saturation and confirming stoichiometry. This stoichiometry was further confirmed by a Job plot constructed at 650 nm (Figure 2C) (see Section 2.2.1 for details). The maximum of the plot occurs at a metal–chelator ratio of 1:1, confirming that the 1:1 complex [SUA–Cu^2+^] is predominantly formed in solution. To calculate the stability constant, the titration curve (Figure 2B) was fitted with the function derived by solving Equations (11)–(14). Given the 1:1 stoichiometry, Equations (7)–(10) for absorbance, stability constant, and mass balance reduce to:

(11) D=[ML]

(12) $$K=\frac{\left[ML\right]}{\left[M\right][L]}$$

(13) $${\left[M\right]}_{0}=[M]+\left[ML\right]$$

(14) $${\left[L\right]}_{0}=[L]+\left[ML\right]$$

From the fit of the experimental data (Figure 2B) using Equations (11)–(14), the stability constant of the [SUA–Cu^2+^] complex was determined to be K = (9.9 ± 0.5) × 10^6^ M^−1^, and the molar extinction coefficient ε at 650 nm was found to be 100 ± 5 M^−1^·cm^−1^.

The complexation of SUA with Fe^3+^ was also studied in a neutral medium. Under these conditions, coordination of Fe^3+^ with hydroxide ions and formation of light-scattering microheterogeneous species are known to occur [40]. In order to obtain the true absorption spectra of SUA–Fe^3+^ chelate complexes, a digital baseline correction was performed. The far-visible and near-IR range (600–900 nm) was selected as an analytical blind zone where the absorbance of both free Fe^3+^ and its SUA complexes is strictly zero. The scattering contribution in this zone was approximated according to a previous method [41], as follows:

(15) $$D\left(\lambda \right)=a\frac{1}{\lambda^{2}}+b$$

**Figure 3:**
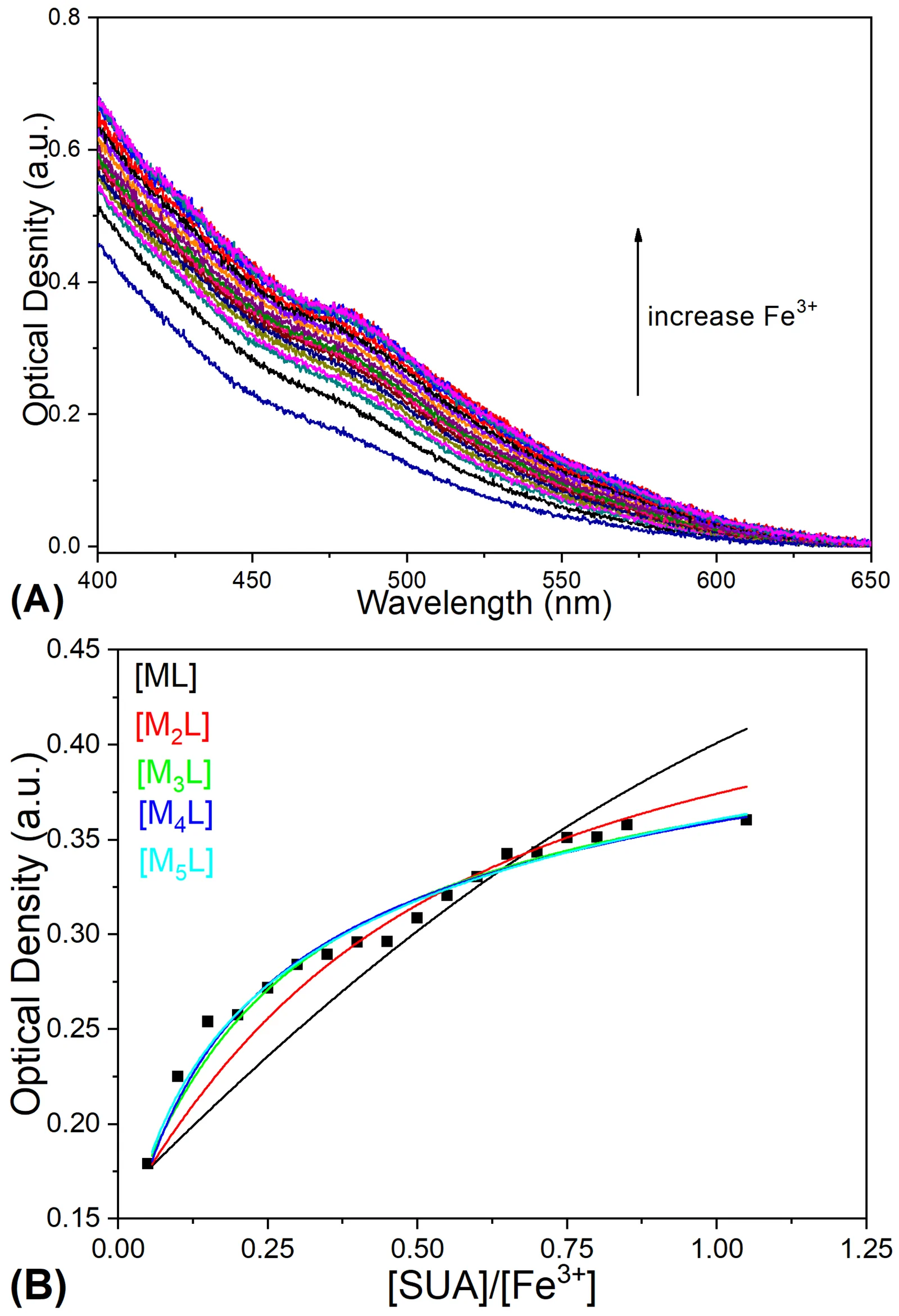

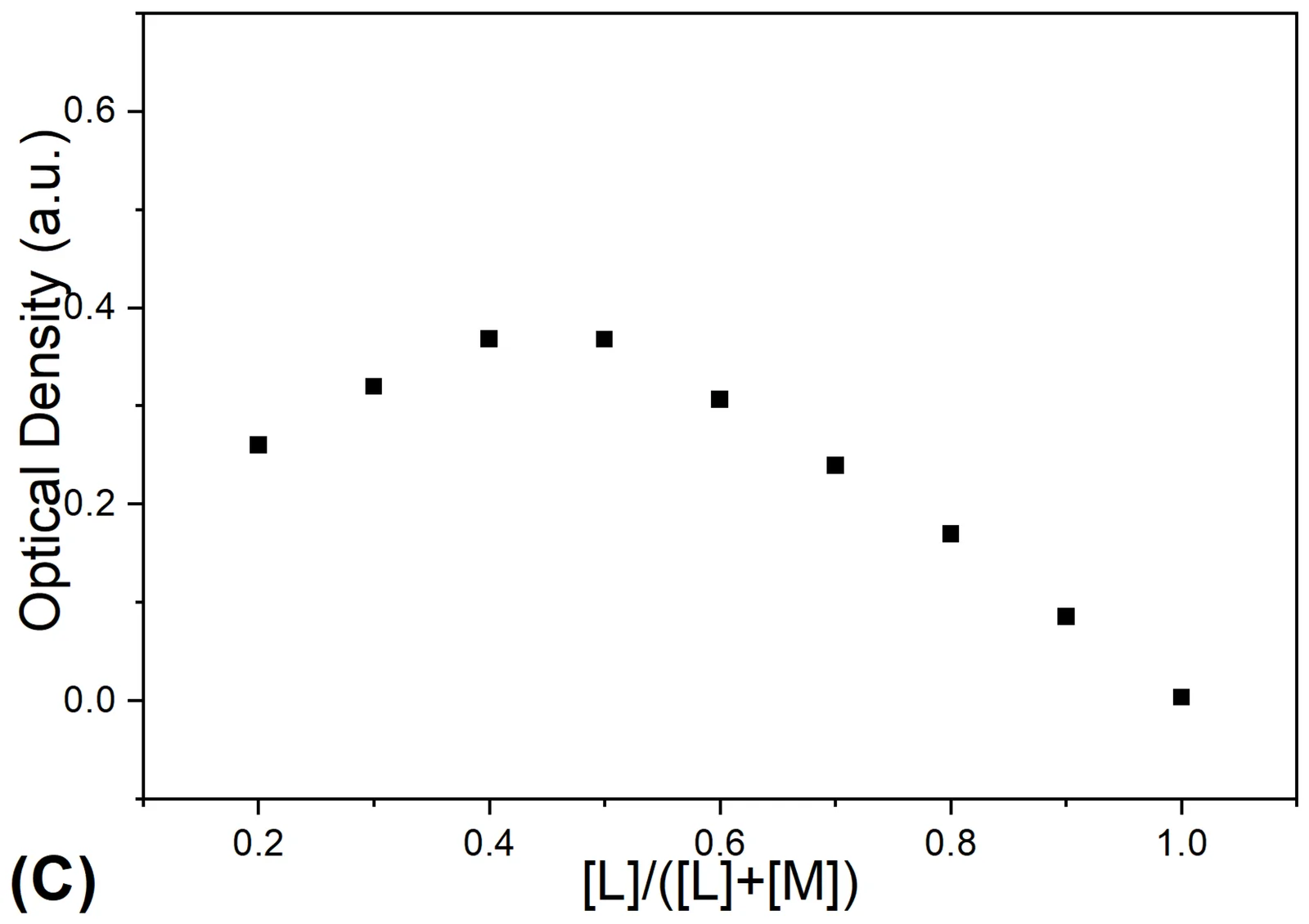
(**A**) UV-Vis absorbance spectra of SUA solutions (0–1.05 mM) in the presence of 1 mM Fe(NO_3_)_3_, after correction for light scattering by particles. (**B**) Absorbance of SUA solutions (0–1.05 mM) in the presence of 1 mM Fe(NO_3_)_3_ at 475 nm after scattering correction, plotted as a function of the [SUA]/[Fe^3+^] ratio and corrected for the colloidal iron fraction. The solid curves represent approximations obtained from Equations (20)–(23) for different values of n. (**C**) Job plot (continuous variation diagram) for SUA–Fe^3+^ mixtures after scattering correction at 475 nm. Total concentration of SUA and Fe^3+^ was 1 mM. All experiments were conducted in 0.1 M HEPES buffer (pH 7.0, adjusted with KOH) at 25 °C.

Parameters a and b were optimized by nonlinear least-squares regression using the curve_fit routine (SciPy library, Python 3) over the 600–900 nm range. Subtraction of the scattering contribution yielded corrected absorption spectra (Figure 3A). The fraction of iron present as scattering particles (colloidal hydroxide) was quantified using an external turbidity calibration method. For this purpose, the spectrum of a blank Fe^3+^ solution was first recorded at a given concentration (C_Fe_blank_ = 5.0 mM) in the absence of chelator at approximately pH 7. After mathematical subtraction of the scattering background for the blank experiment at a fixed reference wavelength of λ = 800 nm, the specific scattering coefficient of the system was calculated as follows:

(16) $$K_{scat}=\frac{D_{Fe\_blank}(800)}{C_{Fe\_blank}}$$

For the working solutions in the titration series, the analytical concentration of colloidal iron (C_Fe_ppt_) at each point was calculated in proportion to the local background intensity at 800 nm, as follows:

(17) $$C_{Fe\_ppt(i)}=\frac{D_{i}(800)}{K_{scat}}$$

For Fe^3+^, the stability and molar extinction constants of the SUA complexes were determined from the absorbance dependence at 475 nm after subtraction of the light-scattering contribution due to particulate species, plotted as a function of the ligand concentration normalized to the fraction of non-colloidal iron (Figure 3B):

(18) $$\left[{Fe}^{3+}\right]=C_{Fe\_total}-C_{Fe\_ppt(i)}$$

The shape of the resulting curve suggests the presence of multiple complex species with different stoichiometries. To test this hypothesis, we constructed a background-corrected Job plot at 475 nm (Figure 3C). The plot did not exhibit a sharp maximum, confirming the coexistence of several stoichiometries. To obtain a reliable fit of the data, various binding models were tested. It was found that the accuracy of the model improves with increasing metal fraction in the stoichiometry of the complex: [ML], [M_2_L], [M_3_L], [M_4_L], [M_5_L] (Figure 3B). It should be noted that in these models, the chelator does not coordinate individual metal ions, but rather a polymeric species. This is evidenced by a sharp increase in the absorbance of the solution at low ligand concentrations. With a further increase in the SUA concentration, the increase in the absorbance of the solution at the absorption wavelength of the complex slows down. We assume that with an increase in the proportion of ligand in the solution, high-order polymers disintegrate into polymers of lower order down to [ML]. This hypothesis is supported by previous reports indicating that iron hydroxo complexes in neutral media readily undergo polymerization, forming hydroxypolymers [Fe_m_(OH)_n_]^(3m−n)+^, where m, n ≥ 2 [42]. Thus, the complexation models are presented in the form of Equation (19), and Equations (7)–(10) reduce to Equations (20)–(23).

(19) $${Fe}_{n}+SUA↔[{Fe}_{n}SUA]$$

(20) $$D=\left[M_{n}L\right]+[M_{n}]$$

(21) $$K=\frac{\left[M_{n}L\right]}{\left[M_{n}\right][L]}$$

(22) $${\left[M\right]}_{0}=n[M_{n}]+n\left[M_{n}L\right]$$

(23) $${\left[L\right]}_{0}=[L]+\left[M_{n}L\right]$$

In this situation, we cannot accurately calculate individual stability constants for each of the polynuclear complexes formed in solution. Instead, we provide an upper estimate for the apparent stability constant per iron ion (K’_app_), which is <10^4^ M^−1^.

**Figure 4:**
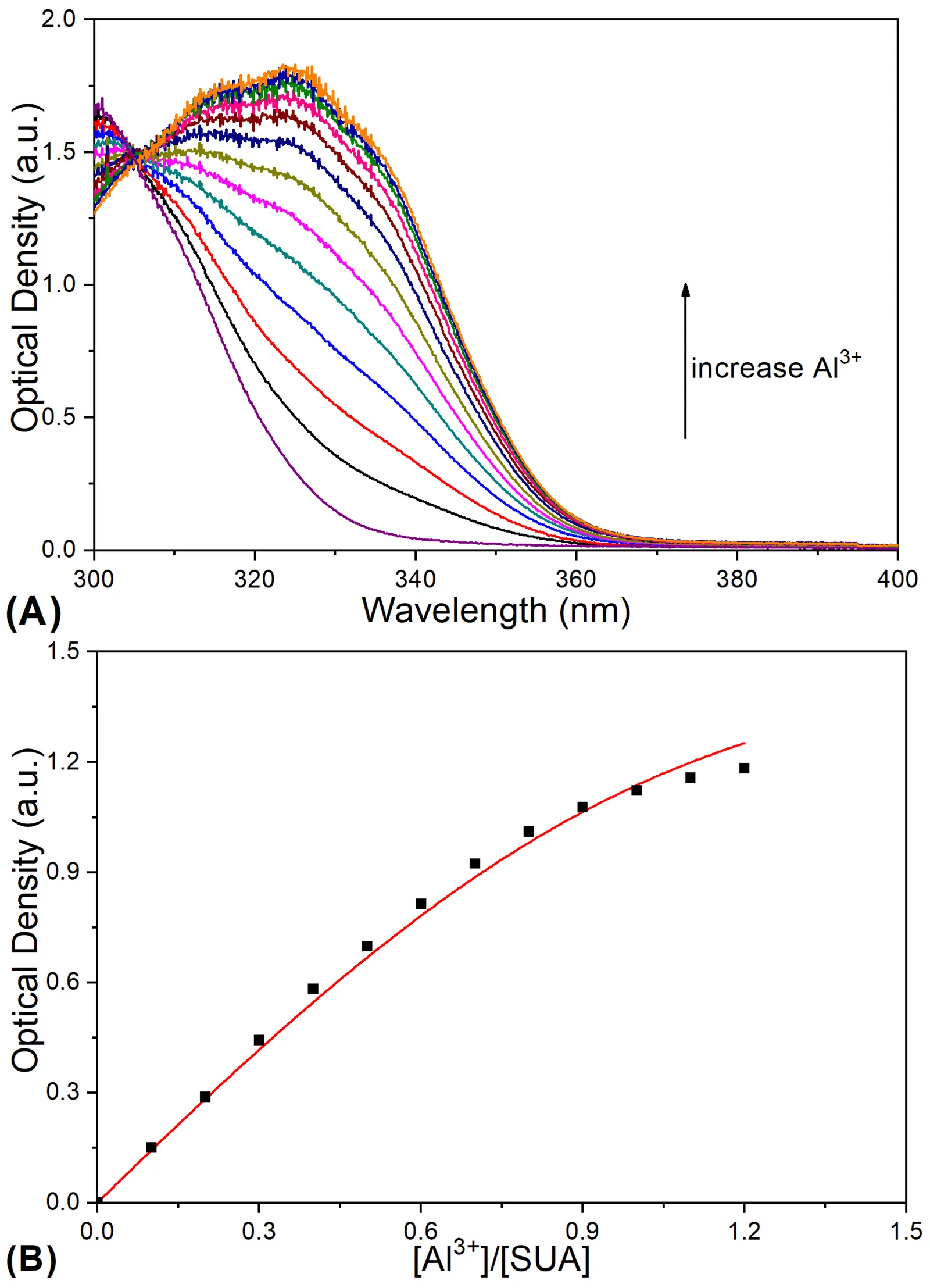

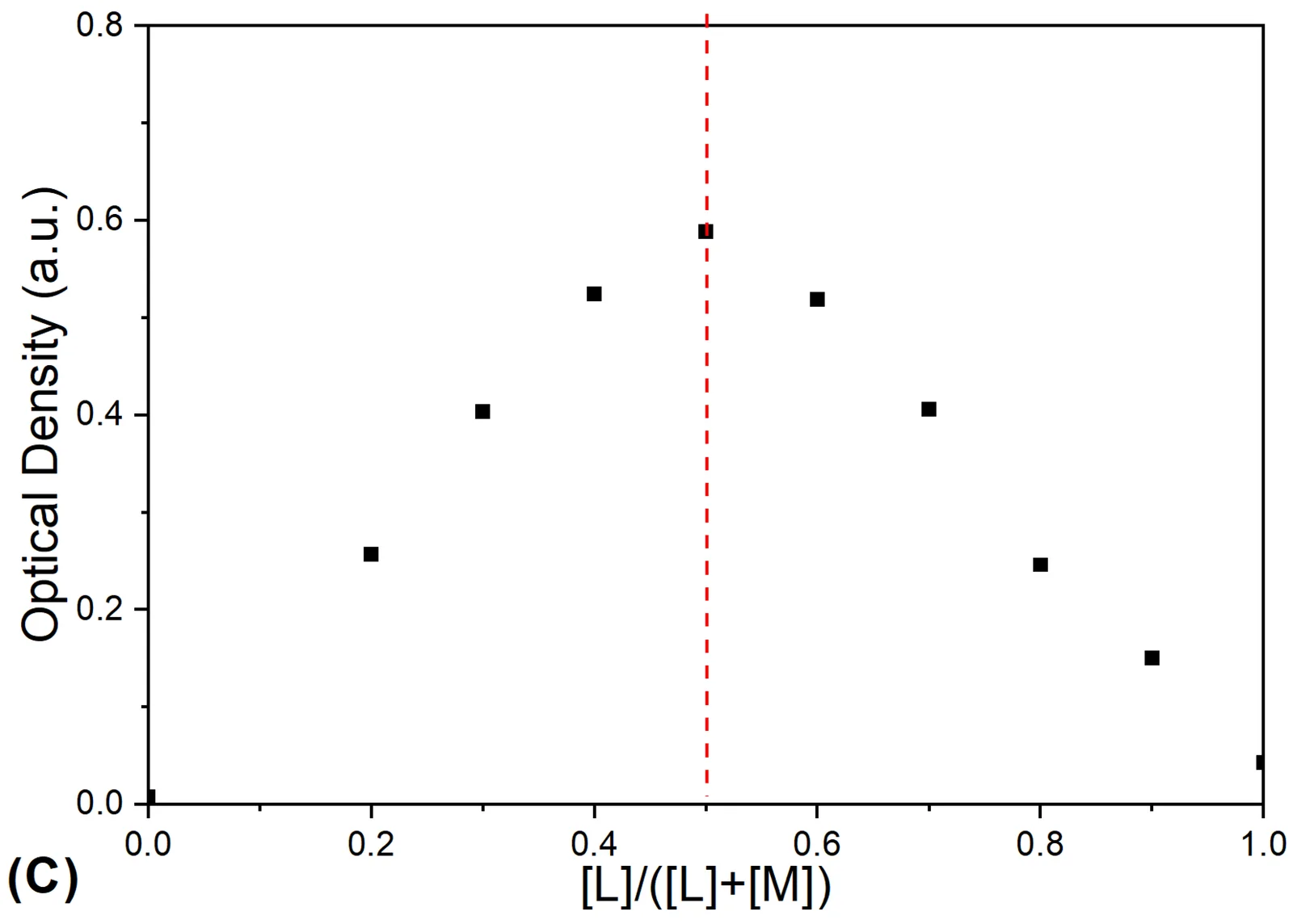
(**A**) UV-Vis absorbance spectra of AlCl_3_ solutions (0–1.2 mM) in the presence of 0.5 mM SUA. (**B**) Absorbance of AlCl_3_ solutions (0–1.2 mM) in the presence of 0.5 mM SUA at 340 nm, at the characteristic wavelength after scattering correction, plotted as a function of the [Al^3+^]/[SUA] ratio. The solid red curve represents the fit obtained from Equations (11)–(14). (**C**) Job plot (continuous variation diagram) for SUA–Al^3+^ mixtures after scattering correction at 340 nm. Total concentration of SUA and Al^3+^ was 0.5 mM. The red dashed lines indicate the maximum for the 1:1 complex. All experiments were conducted in 0.1 M HEPES buffer (pH 5.5, adjusted with KOH) at 25 °C.

In the case of studying the complexation of SUA with Al^3+^ ions, it was not possible to optimize experimental conditions at pH 7 due to the significant predominance of hydroxylation of aluminum ions over their coordination by SUA molecules [43,44]. Experimentally, it was possible to establish that the chelation process begins to predominate over hydroxylation only at pH 5.5. Thus, under these conditions, to calculate the stability constant of the chelate complex of SUA with Al^3+^ ions, a series of optical spectra were recorded with a fixed concentration of SUA and a variable concentration of aluminum salt (Figure 4A). The presence of an isosbestic point in the figure indicates the predominance of one type of complex in the solution.

To determine the stability constant of the SUA complex with Al^3+^, the absorbance of SUA solutions was plotted at various AlCl_3_ concentrations as a function of the [Al^3+^]/[SUA] ratio (Figure 4B). The curve reaches saturation only slowly after a 1:1 ratio, suggesting both this stoichiometry and a low stability constant.

To test this hypothesis, we constructed a Job plot (Figure 4C). The maximum occurs at a mole fraction of 0.5, corresponding to a 1:1 stoichiometry; however, the flat shape of the curve further supports the low stability constant of the complex [36].

The function used to fit the data in Figure 4B was therefore based on a 1:1 binding model (Equations (11)–(14)). The fit yielded a stability constant for the [Al^3+^–SUA] chelate complex of (1.40 ± 0.1) × 10^4^ M^−1^ and a molar extinction coefficient ε at 340 nm of 3500 ± 50 M^−1^·cm^−1^. The calculated stability and extinction constants for the SUA complexes with Fe^3+^, Cu^2+^, and Al^3+^ in neutral medium are summarized in Table 1.

The results of this study demonstrate that although SUA chelates Fe^3+^, Cu^2+^, and Al^3+^ ions, its stability constants with these metal ions are lower under neutral-pH conditions than in acidic media [11,13]. This finding is consistent with literature data showing a decreasing trend in stability constants with increasing pH [13,45]. Furthermore, it should be noted that the stoichiometry of SUA chelate complexes in neutral solutions shifts toward a higher metal-to-chelator ratio. This phenomenon can be attributed to metal hydrolysis, i.e., competition between hydroxide ions and SUA for incorporation into the inner coordination sphere. In the case of Fe^3+^ ions, it was not possible to select an appropriate binding model (Figure 3B). This is likely due to the more complex stoichiometry arising from the polymerization of Fe^3+^ hydroxides in neutral media, as well as the presence of multiple donor atoms in SUA, which potentially afford several modes of metal ion coordination. Thus, the calculated stability constants characterize SUA as a moderate-affinity chelator, comparable to endogenous ligands such as lactate, malate, succinate, and certain amino acids [46,47]. The relatively low affinity for Al^3+^ suggests that SUA is unlikely to significantly affect aluminum metabolic pathways under physiological conditions, although long-term accumulation cannot be entirely excluded.

### 3.2. Coordination of SUA with Fe^2+^ and Cu^+^

**Figure 5:**
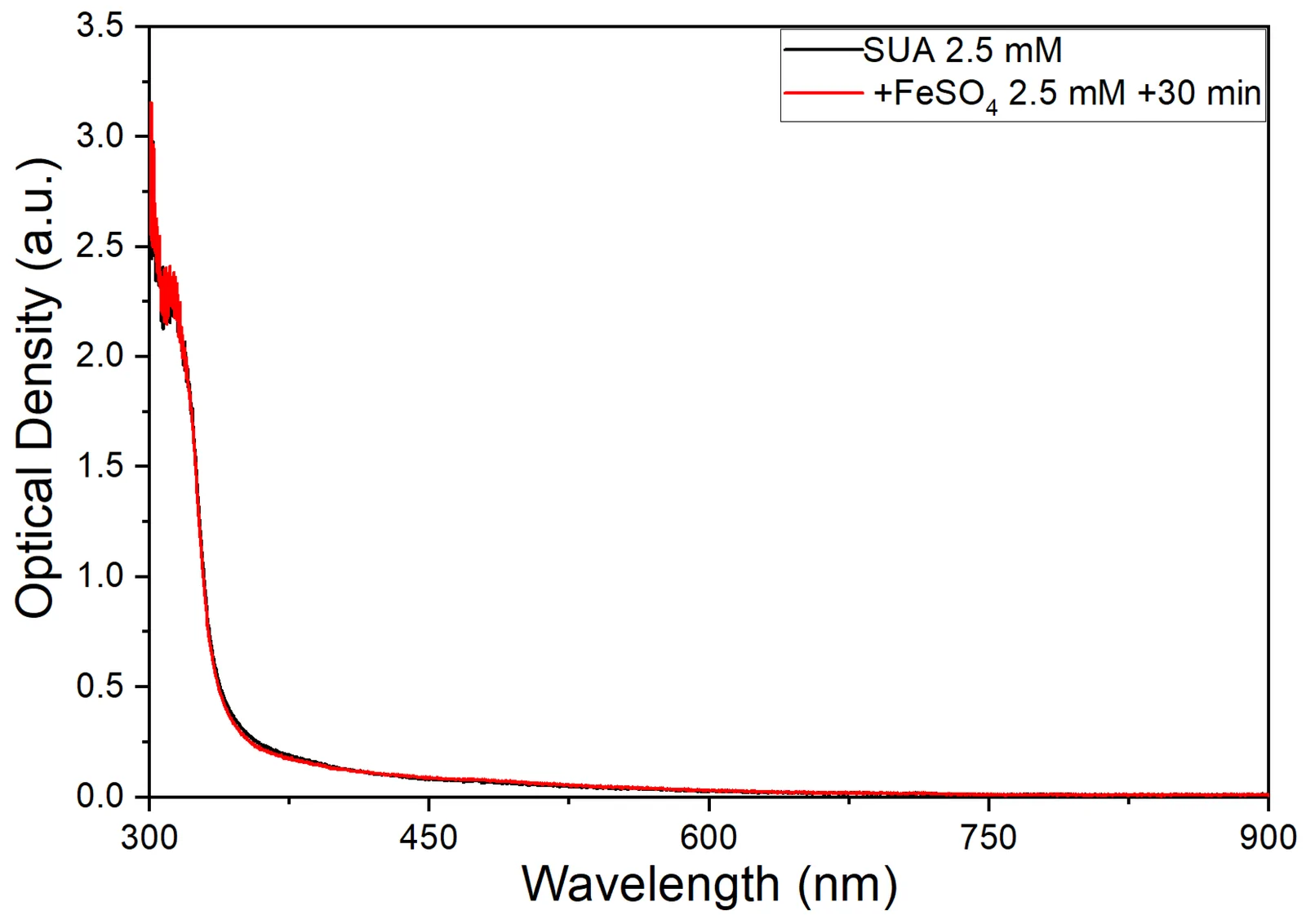
UV-Vis absorbance spectra of a 2.5 mM SUA solution before (black line) and 30 min after (red line) addition of 2.5 mM FeSO_4_. All experiments were conducted in 0.1 M HEPES buffer (pH 7.0, adjusted with KOH) at 25 °C.

In addition to the formation of SUA chelate complexes with oxidized iron and copper ions, we attempted to detect a complex of SUA with Fe^2+^ (Figure 5). However, no changes in the UV-Vis absorption spectrum were observed upon addition of FeSO_4_ to the SUA solution, indicating that SUA most likely does not form stable complexes with Fe^2+^. This finding is consistent with previous reports on other salicylates, for which complex formation was not detected either [48,49].

Complexation of SUA with Cu^+^ could not be observed due to the rapid disproportionation of Cu^+^ to Cu^2+^ and metallic copper (Equation (24)), which causes rapid turbidity and formation of a precipitate [39,50].

(24) $${2Cu}^{+}\to {Cu}^{2+}+{Cu}^{0}$$

Importantly, while no apparent complexation was observed for Fe^2+^ via UV-Vis spectroscopy and Cu^+^ could not be reliably evaluated due to disproportionation, these observations qualitatively point toward a higher affinity of SUA for the more oxidized forms of these metals.

### 3.3. Comparative Evaluation of the Chelating Activity of Salicyluric Acid and Deferiprone

**Figure 6:**
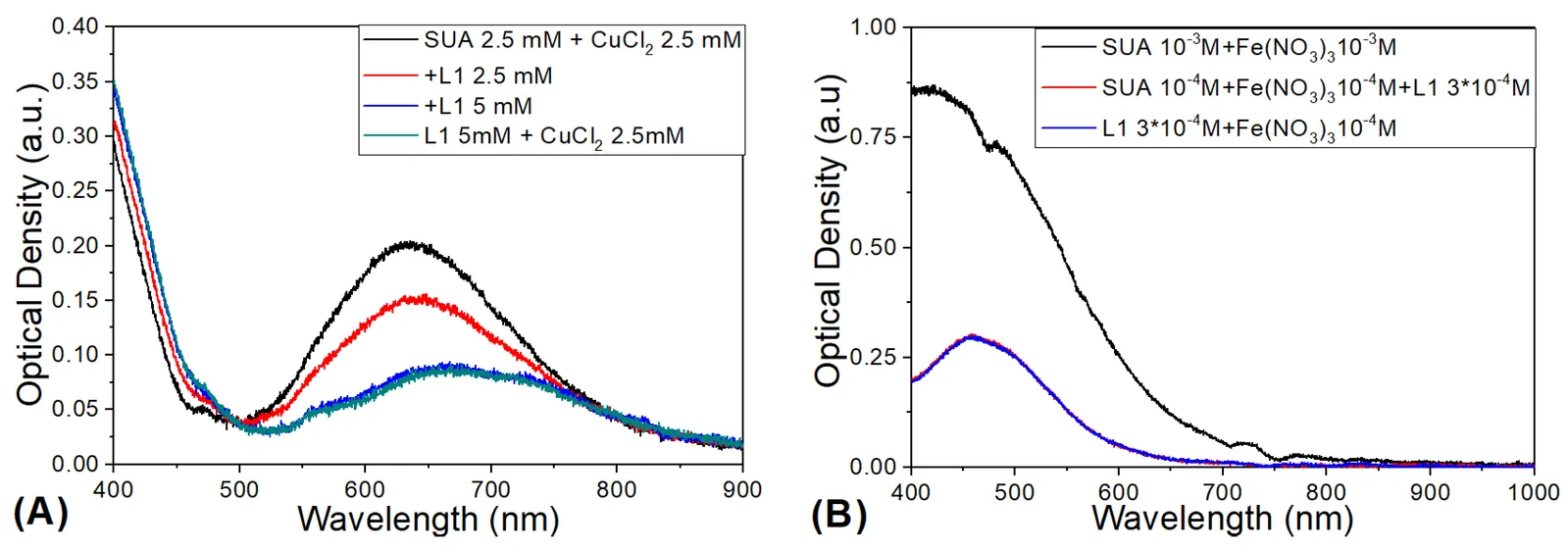
(**A**) UV-Vis absorbance spectra of a mixture of 2.5 mM SUA and 2.5 mM CuCl_2_ in the absence (black) and presence of 2.5 mM (red) or 5 mM (blue) L1. Control: spectrum of a mixture of 5 mM L1 and 2.5 mM CuCl_2_ (green). (**B**) UV-Vis absorbance spectra of a mixture of 1 mM SUA and 1 mM Fe(NO_3_)_3_ in the absence (black) and presence of 0.3 mM L1 after 10-fold dilution. All experiments were conducted in 0.1 M HEPES buffer (pH 7.0, adjusted with KOH) at 25 °C.

To assess the relative chelating efficiency of SUA, competition experiments with Fe^3+^ and Cu^2+^ were performed in the presence of L1 (Figure 6). For Cu^2+^, addition of L1 to the solution caused the disappearance of the band assigned to the SUA–Cu^2+^ complex and the appearance of a band corresponding to [L1_2_Cu], as confirmed by a control experiment (Figure 6A). Thus, ligand exchange in the coordination sphere also occurs for Cu^2+^, as evidenced by the large difference in stability constants: log K for SUA–Cu (6.9) versus that for L1–Cu (up to 19.2, depending on conditions) [24].

Similarly, UV-Vis data showed that addition of L1 to the SUA–Fe^3+^ complex caused a shift from the band characteristic of SUA–Fe^3+^ to that of the [L1_3_Fe] complex (Figure 6B), confirming displacement of SUA by L1 and formation of the extremely stable L1–Fe^3+^ complex (log K ≈ 35) [51]. It should be noted that the absorption band of the L1–Cu^2+^ complex (500–900 nm, ε_max_ ≈ 20 M^−1^·cm^−1^) is characteristic of a d–d electronic transition [52].

### 3.4. The Effect of Ascorbic Acid on Chelate Complexes of SUA with Iron and Copper Ions

**Figure 7:**
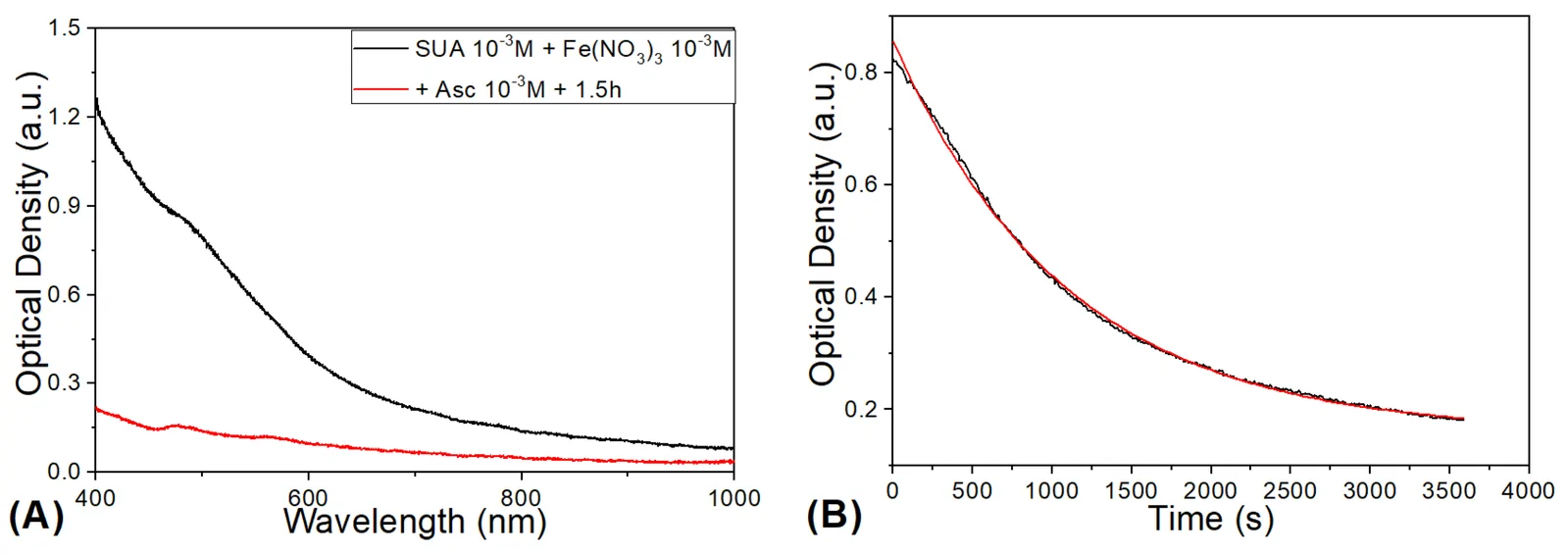

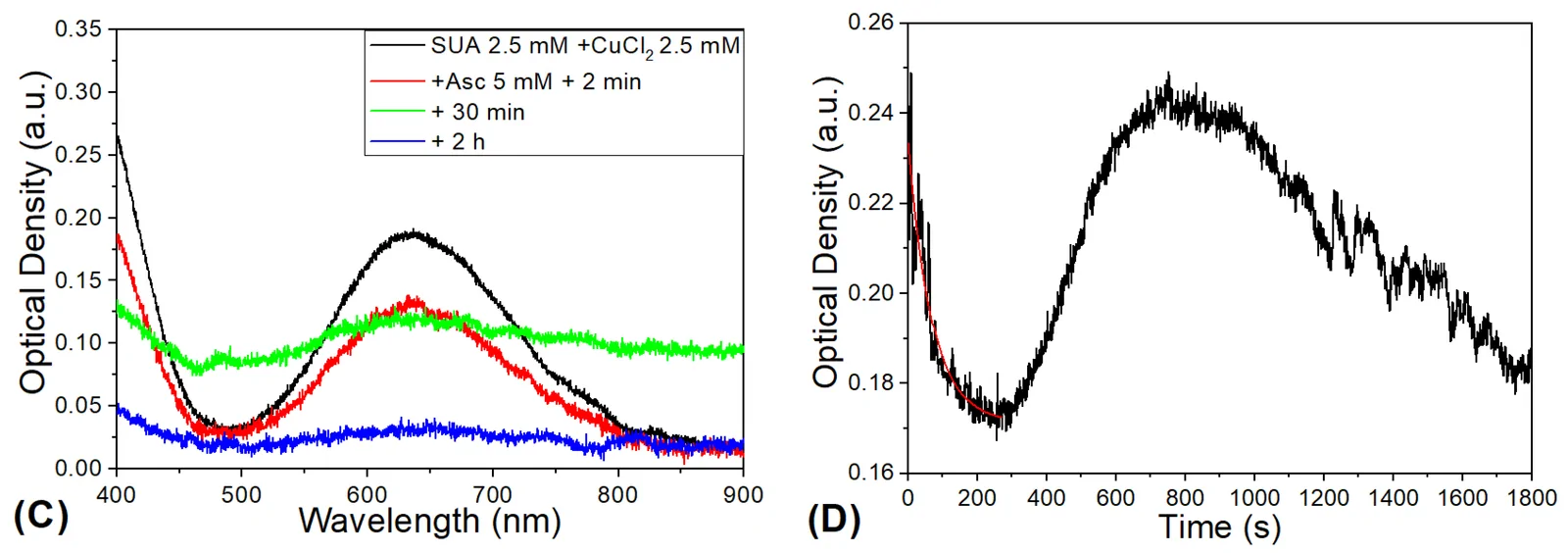
(**A**) UV-Vis absorbance spectra of a solution of 1 mM SUA and 1 mM Fe(NO_3_)_3_ before (black) and 1.5 h after (red) addition of 1 mM Asc. (**B**) Kinetics of absorbance at 475 nm for a solution containing 1 mM SUA, 1 mM Fe(NO_3_)_3_, and 1 mM Asc (black), with the best-fit monoexponential curve (red) obtained using the Levenberg–Marquardt algorithm (y = y_0_ + A·e^−^ᵏᵗ). (**C**) UV-Vis absorbance spectra of a solution of 2.5 mM SUA and 2.5 mM CuCl_2_ before (black) and 2 min (red), 30 min (green), and 2 h (blue) after addition of 5 mM Asc. (**D**) Kinetics of absorbance at 630 nm for a solution containing 2.5 mM SUA, 2.5 mM CuCl_2_, and 5 mM Asc (black), with the best-fit monoexponential curve (red) over the 0–270 s range (y = y_0_ + A·e^−^ᵏᵗ; Levenberg–Marquardt algorithm). All solutions were prepared in 0.1 M HEPES buffer (pH 7.0, adjusted with KOH) at 25 °C.

The effect of Asc on the stability of SUA chelate complexes with Fe^3+^ and Cu^2+^ was also investigated under similar conditions. Addition of Asc to the SUA–Fe^3+^ complex caused a gradual decrease in the absorption band, which had virtually disappeared after 1.5 h (Figure 7A). The kinetics of this process, monitored by the absorbance at 475 nm (Figure 7B), were well described by a monoexponential function with an apparent rate constant of (9.13 ± 0.05) × 10^−4^ s^−1^.

The above UV-Vis absorbance studies suggest that the SUA–Fe^3+^ complex dissociation is attributed to electron transfer from ascorbic acid to the complexed Fe^3+^, yielding Fe^2+^ (Equation (1)).

More complex behavior was observed upon addition of Asc to the SUA–Cu^2+^ complex (Figure 7C,D). The absorbance initially decreased rapidly (within the first 270 s), with concomitant disappearance of the complex band. This was followed by a turbidity-induced increase in absorbance over the next 550 s and a final decrease due to precipitation.

This behavior can be explained by two processes. First, as with Fe^3+^, electron transfer from Asc to complexed Cu^2+^ yields Cu^+^ (Equation (2)). This initial phase was also fitted with a monoexponential function, yielding an apparent rate constant of (1.44 ± 0.08) × 10^−2^ s^−1^. However, unlike Fe^2+^, the resulting Cu^+^ is unstable in aqueous solution and undergoes disproportionation (Equation (24)), leading to turbidity due to metallic copper nanoparticles, which subsequently aggregate and precipitate together with Cu(OH)_2_ [39,50].

Thus, competition experiments with L1 unequivocally demonstrated that this strong chelator displaces SUA from the coordination sphere of both Fe^3+^ and Cu^2+^, confirming the significantly higher stability of L1–metal complexes. This observation is consistent with the thermodynamic preference of hexadentate hydroxypyridinone ligands over bidentate salicylates [53,54].

### 3.5. The Effect of Ascorbic Acid on the Peroxidation of Linoleic Acid Micelles with the Participation of Iron and Copper Ions in the Presence of Salicyluric Acid

Further studies were undertaken to investigate the effect of Asc on the redox activity of SUA chelate complexes with Fe^3+^ and Cu^2+^. As a model system, we used linoleic acid (LA) micelles formed in aqueous solution at neutral pH [55]. Experimental details are provided in Section 2.2.2.

This system serves as a model for peroxidation of unsaturated lipids in biological membranes. The free-radical chain oxidation of unsaturated lipids is well documented [56,57] and proceeds via three stages: initiation, propagation, and termination. This process has been described in detail in previous studies [24,58]. In this study, the focus was on the initiation and propagation steps, which involves hydrogen abstraction from a lipid molecule by a hydroxyl radical to yield a carbon-centered lipid radical:

(25) $$LH+\dot{OH}\to \dot{L}+H_{2}O$$

**Figure 8:**
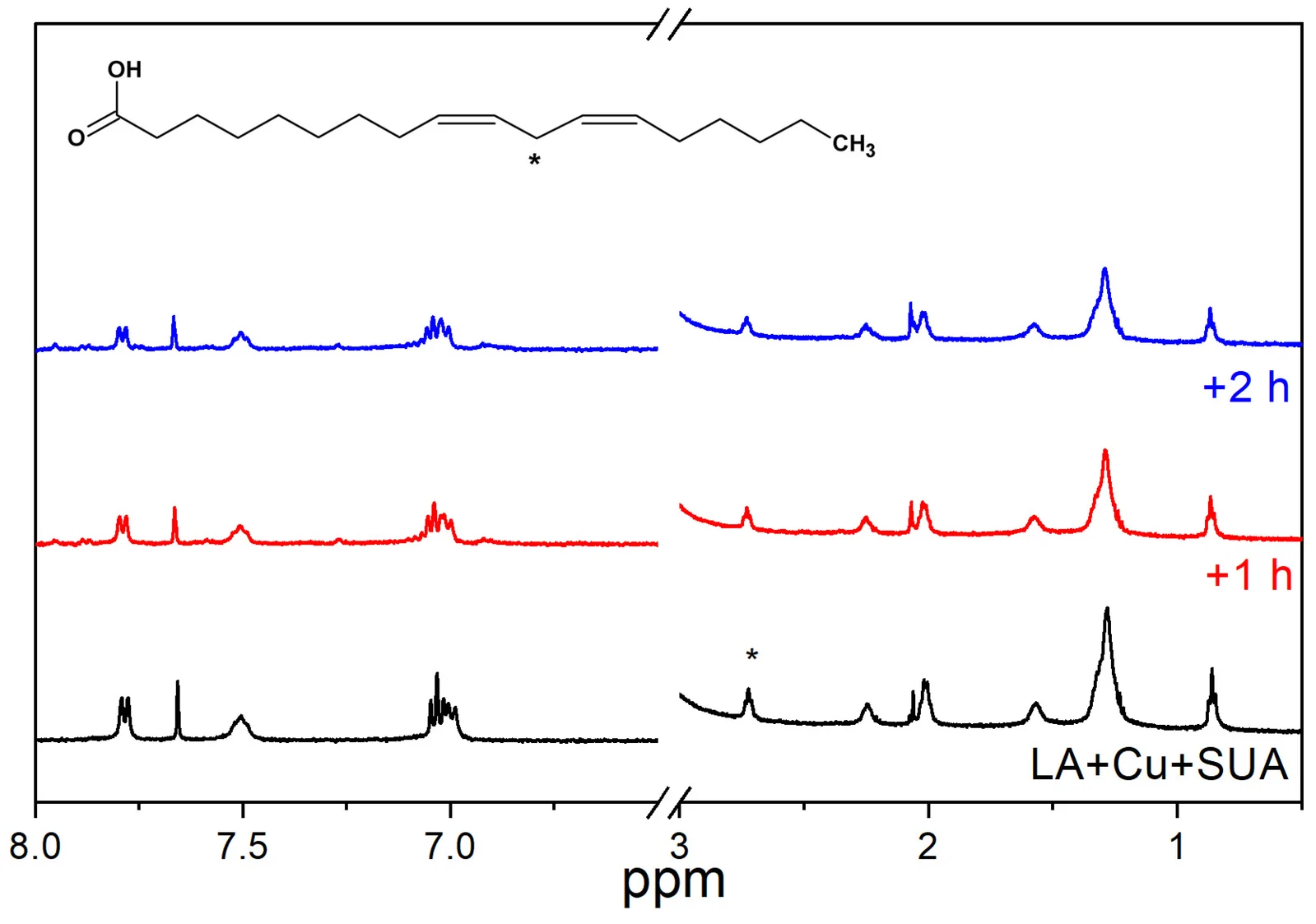
Section of the ^1^H NMR spectra of linoleic acid (LA) micelles (3.5 mM) in a solution containing 1 mM SUA and 0.1 mM CuCl_2_, recorded immediately after addition of 0.5 M H_2_O_2_ (black), and after 1 h (red) and 2 h (blue). All solutions were prepared in 10 mM Na-PIPES buffer (pH 6.8) at 303 K. The bis-allylic proton resonance of LA is marked with an asterisk (*).

Hydroxyl radicals, which trigger lipid peroxidation, are formed as a result of the Fenton reaction (Equations (3) and (5)). For unsaturated lipids such as LA, the primary target of hydroxyl radical attack is the bis-allylic proton adjacent to the double bonds (Figure 8) [59]. Thus, by modulating ·OH generation via coordination of transition metal ions, we can influence the initiation step and thereby affect the rate of lipid consumption.

To assess the effect of metal ion coordination on LA peroxidation, a series of ^1^H NMR spectra were recorded in the absence and presence of SUA and Asc. For these NMR experiments, Na-PIPES buffer was used instead of HEPES. This monosodium-substituted zwitterionic buffer also belongs to the Good buffer family, but unlike HEPES does not produce signals in the 2.7 ppm region, where the bis-allylic proton of LA resonates. This difference allows the monitoring of changes in lipid concentration during oxidation [60].

As an example, Figure 8 shows the ^1^H NMR spectra of LA micelles during copper-mediated peroxidation in the presence of SUA. The integrated intensity of the bis-allylic proton signal decreases over time, while that of SUA protons (7–8 ppm) also changes, indicating degradation of both the lipid and the chelator. Furthermore, it is clear that the other signals from LA also decrease. This is likely due to the high ionic strength of the solution, caused by the high Na^+^ content in the buffer [61,62].

**Figure 9:**
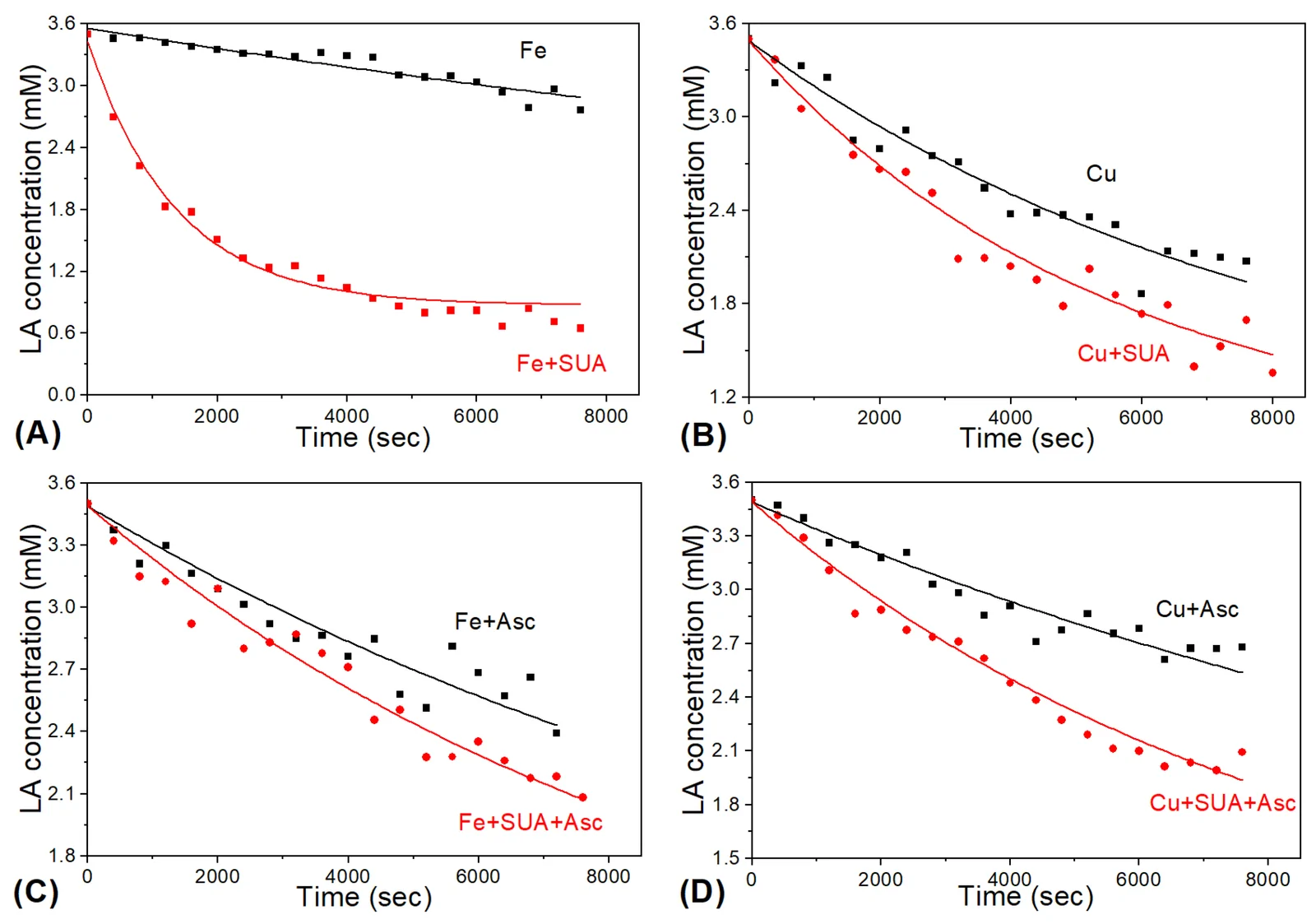
Time dependence of the integrated intensity of the bis-allylic proton signal (2.7 ppm) of linoleic acid (LA) during peroxidation, expressed as the fraction of the initial LA concentration (3.5 mM). (**A**) In the absence (black) and presence (red) of 1 mM SUA with 0.1 mM Fe(NO_3_)_3_. (**B**) In the absence (black) and presence (red) of 1 mM SUA with 0.1 mM CuCl_2_. (**C**) In the absence (black) and presence (red) of 1 mM SUA with 0.1 mM Fe(NO_3_)_3_ and 5 mM Asc. (**D**) In the absence (black) and presence (red) of 1 mM SUA with 0.1 mM CuCl_2_ and 5 mM Asc. Each curve was fitted with an exponential function. All experiments were carried out in D_2_O using 10 mM Na-PIPES buffer at 303 K and pH 6.8. The peroxidation reaction was initiated by addition of 0.5 M H_2_O_2_.

The time dependence of the integrated intensity of the bis-allylic proton signal of LA for systems containing Fe^3+^/Cu^2+^, SUA, and/or Asc in various combinations was constructed based on the data obtained above (Figure 9). The intensity was converted to LA concentration by calibrating the first time point against the initial LA concentration (3.5 mM). LA precipitation was taken into account by conducting control experiments in the absence of H_2_O_2_ and Asc.

It is worth noting that control experiments were also conducted to study LA oxidation in the presence of Cu^2+^ and Fe^3+^ in the absence of H_2_O_2_. It was found that the LA oxidation rate under these conditions was more than two orders of magnitude lower than in the presence of H_2_O_2_. Separate experiments were also conducted with FeSO_4_ to study LA peroxidation. The results were consistent with those obtained with Fe^3+^.

To quantify the effects of metal coordination and the redox agent Asc on the peroxidation of LA micelles, we fitted the time dependence of the integrated LA intensity with an exponential decay function (Figure 9) to obtain the effective rate constants of LA decay. To accurately compare the series of experiments, a simultaneous (global) nonlinear regression analysis was used. The minimum of the global function of the residual vectors was found using the least-squares method. Calculations were performed using a custom-written Python 3 script. The calculated values are summarized in Table 2.

Individually, both SUA and Asc accelerated the rate of LA peroxidation in the presence of iron ions. However, for copper ions, the presence of Asc slowed the degradation of LA within micelles. Furthermore, a comparison of the decay constants for the SUA-containing system versus the SUA + Asc mixture revealed a decrease in the oxidation rate.

Both Fe^3+^ and Cu^2+^ complexes of SUA accelerated lipid oxidation, as evidenced by the accelerated decay of the integrated signal intensity from the bis-allylic protons of LA (Figure 9A,B). At first glance, this finding appears to contradict our previous study on the membrane-tropic properties of SUA and its redox activity in the peroxidation reaction [59], in which it was suggested that SUA does not affect the rate of LA peroxidation. However, the discrepancy in results can be explained by the choice of buffer: in the present work, we used the organic buffer Na-PIPES, which does not coordinate transition metal ions [37], whereas in our previous study we employed phosphate-buffered saline (PBS), which can be incorporated into the inner coordination sphere of the metal ion [63,64] and compete with SUA for binding, likely affecting the rate of LA oxidation in our earlier work [59]. In the case of SUA and its moderate stability constants to iron and copper ions, it was found that the buffer is able to influence the redox activity of SUA in the peroxidation reaction of LA micelles due to competitive binding with iron and copper ions. This is confirmed by independent studies, which also show that PBS affects the redox activity of dissolved chelating agents [65,66]. We believe that not only do the results obtained in this work not contradict our previous findings [59], but they also clarify and complement them. PBS is closer to biological conditions than Na-PIPES due to the presence of counterions. Therefore, the conclusions on the redox activity of SUA presented in the article [59] more accurately describe the behavior of SUA in biological systems. In this same study, we discovered a more subtle effect in the absence of competitive coordination of transition metal ions.

Nevertheless, this does not answer the question regarding the nature of the pro-oxidant activity of SUA. On the contrary, one might expect SUA to slow down the lipid peroxidation reaction, as literature data on salicylates suggest that they are predominantly antioxidants [67,68]. It is plausible that in a heterogeneous non-physiological system, the redox potentials of the complexed metal ions are altered, thereby enhancing the efficiency of ROS generation (Equations (3)–(6)).

A more complex pattern was observed when Asc was added to preformed SUA–metal complexes in a homogeneous medium. In the SUA–Fe^3+^ system, Asc rapidly reduced Fe^3+^ to Fe^2+^, leading to the dissociation of the chelate complex (Figure 7A). Similarly, in the SUA–Cu^2+^ system, Asc reduced Cu^2+^ to Cu^+^, followed by the disproportionation of Cu^+^ to Cu^0^ and Cu^2+^, which caused turbidity and precipitation (Figure 7C). Critically, the combined presence of SUA and Asc in the LA peroxidation system did not produce an additive pro-oxidant effect: rather, a slowdown in LA oxidation was observed compared to systems containing SUA or Asc alone (Figure 9). This suggests that the heterogeneity of the system in this case shifts the reaction pathways, reducing the ability of Asc to reduce Fe^3+^ and Cu^2+^ ions and highlighting the capacity of Asc to scavenge ROS [69]. These observations may have biological relevance, as aspirin users often take also vitamin C (Asc), and it may be possible that the interplay between SUA and Asc could modulate oxidative stress in vivo.

Further studies are warranted to explore the biological effects of this aspirin metabolite (SUA) on redox activity and metal ions, including iron, copper, and other essential metal metabolism and toxicity. Similarly, more studies are warranted on the influence of SUA on xenobiotic metal metabolism and toxicity such as those present in aluminum antacids, radiopharmaceuticals, and environmental pollutants [70,71,72,73,74].

## 4. Conclusions

The chelating and redox properties of salicyluric acid (SUA), the primary metabolite of aspirin, with physiologically relevant transition metal ions (Cu^2+^, Fe^3+^) and a xenobiotic metal (Al^3+^) was studied under neutral pH conditions. It was demonstrated that SUA forms stable 1:1 complexes with Cu^2+^ and Al^3+^. In the case of Fe^3+^, there is no dominant stoichiometry of the complexes due to the polymerization of iron ions during hydroxylation in neutral solutions. It should be noted that while no apparent complexation was detected with Fe^2+^ by UV-Vis spectroscopy and Cu^+^ could not be reliably tested due to disproportionation, these findings tentatively suggest that SUA may favor higher oxidation states of these metals. The determined stability constants (log K = 6.99 for Cu^2+^ and 4.15 for Al^3+^) classify SUA as a moderate-affinity chelator, comparable to endogenous chelators such as various carboxylates and certain amino acids [46,47], but considerably weaker than the clinically used chelator L1. In the case of Fe^3+^, an upper estimate for the apparent stability constant per iron ion is given (K’_app_ < 10^4^ M^−1^). Competition experiments with L1 confirmed the quantitative displacement of SUA from metal coordination spheres, underscoring the superior chelating efficiency of the hydroxypyridone chelator.

The redox activity of SUA complexes was evaluated in a Fenton-initiated lipid peroxidation model using LA micelles. Both Fe^3+^ and Cu^2+^ complexes of SUA accelerated lipid oxidation, indicating a pro-oxidant effect mediated by metal ion-catalyzed radical generation. In the case of Asc, there was an increase in the rate of peroxidation in the presence of iron ions and a decrease in the rate in the presence of copper ions. However, with the combination of SUA and Asc, a net decrease in the peroxidation rate was observed compared to solutions containing SUA. Kinetic analysis of LA peroxidation based on NMR data provided direct evidence that SUA and Asc modulate the initiation stage of the lipid oxidation chain reaction. The exponential decay of the bis-allylic proton signal allowed quantification of apparent LA decay rate constants, revealing that the SUA–Asc combination acts as an inhibitor of peroxidation relative to pure SUA.

Taken together, the results of this study demonstrate that SUA, while a moderate chelator, is redox-active and can influence metal-driven oxidative processes. The competition between SUA and stronger chelators such as L1, as well as the modulation of its activity by Asc, highlights the complexity of metal ion homeostasis in the presence of aspirin and other similar drug metabolites. Furthermore, the shift in stoichiometry toward higher-metal-content complexes at neutral pH, attributed to metal hydrolysis and competition with hydroxide ions, underscores the importance of considering both coordination chemistry and solution conditions when evaluating the biological effects of drug metabolites. Further studies are warranted to explore the potential synergistic or antagonistic effects of SUA with other dietary or pharmaceutical chelators and antioxidants, the impact on cellular systems, such as essential and xenobiotic metal metabolism, and redox balance, and also in relation to the treatment of different clinical conditions, including chronic aspirin therapy.

## Figures and Tables

**Table 1:** Calculated stoichiometries and stability constants of SUA with Cu^2+^ и Al^3+^ ions in water in neutral pH. In the case of Fe^3+^, the underlying stoichiometry of the complex was not determined, and an upper estimate for the apparent stability constant per iron ion is given.

Metal Ion	Stoichiometry (Metal: Ligand)	Stability Constant (M^−1^)
Cu^2+^	1:1	(9.9 ± 0.5) × 10^6^
Fe^3+^	-	<10^4^
Al^3+^	1:1	(1.40 ± 0.1) × 10^4^

**Table 2:** Effective constants of linoleic acid (LA) decay induced by iron (Fe) and copper (Cu) ions in the presence and in the absence of salicyluric acid (SUA) and ascorbic acid (Asc). The concentrations of SUA, Asc, and metal salts were the same as those shown in Figure 9.

Mixture	Effective Constant of LA Decay (s^−1^) with Fe Ions	Effective Constant of LA Decay (s^−1^) with Cu Ions
LA	(3.8 ± 0.3) × 10^−5^	(1.18 ± 0.04) × 10^−4^
LA + SUA	(7.4 ± 0.5) × 10^−4^	(1.8 ± 0.1) × 10^−4^
LA + Asc	(7.2 ± 0.4) × 10^−5^	(5.9 ± 0.4) × 10^−5^
LA + SUA + Asc	(1.1 ± 0.1) × 10^−4^	(1.0 ± 0.03) × 10^−4^

## Data Availability

The original contributions presented in this study are included in the article. Further inquiries can be directed to the corresponding authors.

## Abbreviations

The following abbreviations are used in this manuscript.

Asc	ascorbic acid
D_2_O	deuterium oxide
HEPES	4-(2-hydroxyethyl)piperazine-1-ethanesulfonic acid (also known as N-(2-hydroxyethyl)piperazine-N′-(2-ethanesulfonic acid))
L1	deferiprone (1,2-dimethyl-3-hydroxypyrid-4-one)
LA	linoleic acid
Na-PIPES	piperazine-N,N′-bis(2-ethanesulfonic acid) sodium salt (sodium 1,4-piperazinediethanesulfonate)
PBS	phosphate-buffered saline
SUA	salicyluric acid (2-hydroxyhippuric acid)

## References

[1] Werz O., Stettler H., Theurer C., Seibel J. (2024). The 125th Anniversary of Aspirin—The Story Continues. Pharmaceuticals.

[2] Della Bona R., Giubilato S., Palmieri M., Benenati S., Rossini R., Di Fusco S.A., Novarese F., Mascia G., Gasparetto N., Di Monaco A. (2024). Aspirin in Primary Prevention: Looking for Those Who Enjoy It. J. Clin. Med..

[3] Mekaj Y.H., Daci F.T., Mekaj A.Y. (2015). New Insights into the Mechanisms of Action of Aspirin and Its Use in the Prevention and Treatment of Arterial and Venous Thromboembolism. Ther. Clin. Risk Manag..

[4] Visagie J.L., Aruwajoye G.S., van der Sluis R. (2024). Pharmacokinetics of Aspirin: Evaluating Shortcomings in the Literature. Expert Opin. Drug Metab. Toxicol..

[5] Kim J.H., Shim M.J., Lee S.-Y., Oh J., Kim S.H. (2019). Aspirin for Primary Prevention of Cardiovascular Disease. J. Lipid Atheroscler..

[6] Pacifici G.M. (2024). Clinical Pharmacology of Aspirin. Biomed. J. Sci. Tech. Res..

[7] Sidlak A.M., Spadaro A., Koyfman A., Long B. (2025). Acute Salicylate Toxicity: A Narrative Review for Emergency Clinicians. Cureus.

[8] Kontoghiorghes G.J. (2024). The Puzzle of Aspirin and Iron Deficiency: The Vital Missing Link of the Iron-Chelating Metabolites. Int. J. Mol. Sci..

[9] Kontoghiorghes G.J. (2025). New Insights into Aspirin’s Anticancer Activity: The Predominant Role of Its Iron-Chelating Antioxidant Metabolites. Antioxidants.

[10] Alaklabi A.A., Altowaim A.S., Alqahtani H.S., Moammar A.A.B., Alturki A.T., Muammar R.A.A. (2025). Prevalence of Iron Deficiency Anaemia in Elderly Aged > 60 Taking Daily Low-Dose Aspirin. J. Aging Res..

[11] Park M.V. (1966). Complex Formation between Iron(III) and Some Substituted Salicylic Acids. J. Chem. Soc. A Inorg. Phys. Theor..

[12] Bermejo Gonzalez E., Daeid N.N., Nolan K.B., Farkas E. (1994). Complex Formation between Transition Metal Ions and Salicylglycine, a Metabolite of Aspirin. Polyhedron.

[13] Pecci J., Foye W.O. (1960). The Avidity of Salicylic, Gentisic, and Salicyluric Acids for Heavy Metal Cations. J. Am. Pharm. Assoc. Sci. Ed..

[14] Kontoghiorghes G.J., Kolnagou A., Kontoghiorghe C.N., Mourouzidis L., Timoshnikov V.A., Polyakov N.E. (2020). Trying to Solve the Puzzle of the Interaction of Ascorbic Acid and Iron: Redox, Chelation and Therapeutic Implications. Medicines.

[15] Fowler A.A., Truwit J.D., Hite R.D., Morris P.E., Dewilde C., Priday A., Fisher B., Thacker L.R., Natarajan R., Brophy D.F. (2019). Effect of Vitamin C Infusion on Organ Failure and Biomarkers of Inflammation and Vascular Injury in Patients with Sepsis and Severe Acute Respiratory Failure: The CITRIS-ALI Randomized Clinical Trial. JAMA.

[16] Dakhale G.N., Chaudhari H.V., Shrivastava M. (2011). Supplementation of Vitamin C Reduces Blood Glucose and Improves Glycosylated Hemoglobin in Type 2 Diabetes Mellitus: A Randomized, Double-Blind Study. Adv. Pharmacol. Sci..

[17] Appiah D., Ingabire-Gasana E., Appiah L., Yang J. (2024). The Relation of Serum Vitamin C Concentrations with Alzheimer’s Disease Mortality in a National Cohort of Community-Dwelling Elderly Adults. Nutrients.

[18] Ma Y., Chapman J., Levine M., Polireddy K., Drisko J., Chen Q. (2014). High-Dose Parenteral Ascorbate Enhanced Chemosensitivity of Ovarian Cancer and Reduced Toxicity of Chemotherapy. Sci. Transl. Med..

[19] Bielski B.H.J., Seib P.A., Tolbert B.M. (1982). Chemistry of Ascorbic Acid Radicals. Ascorbic Acid: Chemistry, Metabolism, and Uses.

[20] Milburn R.M., Vosburgh W.C. (1955). A Spectrophotometric Study of the Hydrolysis of Iron(III) Ion. II. Polynuclear Species. J. Am. Chem. Soc..

[21] Gallard H., De Laat J., Legube B. (1999). Spectrophotometric Study of the Formation of Iron(III)-Hydroperoxy Complexes in Homogeneous Aqueous Solutions. Water Res..

[22] Pham A.N., Rose A.L., Waite T.D. (2012). Kinetics of Cu(II) Reduction by Natural Organic Matter. J. Phys. Chem. A.

[23] Delangle P., Mintz E. (2012). Chelation Therapy in Wilson’s Disease: From d-Penicillamine to the Design of Selective Bioinspired Intracellular Cu(I) Chelators. Dalton Trans..

[24] Timoshnikov V.A., Kobzeva T., Selyutina O.Y., Polyakov N.E., Kontoghiorghes G.J. (2019). Effective Inhibition of Copper-Catalyzed Production of Hydroxyl Radicals by Deferiprone. J. Biol. Inorg. Chem..

[25] Deng J., Ramelli L., Li P.Y., Eshaghpour A., Li A., Schuenemann G., Crowther M.A. (2024). Efficacy of Vitamin C with Fe Supplementation in Patients with Iron Deficiency Anemia: A Systematic Review and Meta-Analysis. Blood Vessels Thromb. Hemost..

[26] Piskin E., Cianciosi D., Gulec S., Tomas M., Capanoglu E. (2022). Iron Absorption: Factors, Limitations, and Improvement Methods. ACS Omega.

[27] Zümreoglu-Karan B. (2006). The Coordination Chemistry of Vitamin C: An Overview. Coord. Chem. Rev..

[28] Kontoghiorghes G.J., Kleanthous M., Kontoghiorghe C.N. (2020). The History of Deferiprone (L1) and the Paradigm of the Complete Treatment of Iron Overload in Thalassaemia. Mediterr. J. Hematol. Infect. Dis..

[29] Nurchi V.M., Crisponi G., Pivetta T., Donatoni M., Remelli M. (2008). Potentiometric, Spectrophotometric and Calorimetric Study on Iron(III) and Copper(II) Complexes with 1,2-Dimethyl-3-Hydroxy-4-Pyridinone. J. Inorg. Biochem..

[30] Timoshnikov V.A., Kobzeva T.V., Polyakov N.E., Kontoghiorghes G.J. (2020). Redox Interactions of Vitamin c and Iron: Inhibition of the pro-Oxidant Activity by Deferiprone. Int. J. Mol. Sci..

[31] Bryliński Ł., Kostelecka K., Woliński F., Duda P., Góra J., Granat M., Flieger J., Teresiński G., Buszewicz G., Sitarz R. (2023). Aluminium in the Human Brain: Routes of Penetration, Toxicity, and Resulting Complications. Int. J. Mol. Sci..

[32] de Souza A.B.F., Kozima E.T., Castro T.d.F., de Matos N.A., Oliveira M., de Souza D.M.S., Talvani A., de Menezes R.C.A., Cangussú S.D., Bezerra F.S. (2023). Chronic Oral Administration of Aluminum Hydroxide Stimulates Systemic Inflammation and Redox Imbalance in BALB/c Mice. Biomed. Res. Int..

[33] Igbokwe I.O., Igwenagu E., Igbokwe N.A. (2019). Aluminium Toxicosis: A Review of Toxic Actions and Effects. Interdiscip. Toxicol..

[34] Gaspari F., Viganò G., Locatelli M., Remuzzi G. (1988). Influence of Antacid Administrations on Aspirin Absorption in Patients with Chronic Renal Failure on Maintenance Hemodialysis. Am. J. Kidney Dis..

[35] Kaniwa N., Ogata H., Aoyagi N., Ejima A. (1981). The Bioavailabilities of Aspirin from an Aspirin Aluminum and an Aspirin Tablet and the Effects of Food and Aluminum Hydroxide Gel. J. Pharmacobio-Dyn..

[36] Renny J.S., Tomasevich L.L., Tallmadge E.H., Collum D.B. (2013). Method of Continuous Variations: Applications of Job Plots to the Study of Molecular Associations in Organometallic Chemistry. Angew. Chem. Int. Ed. Engl..

[37] Pielak G.J. (2021). Buffers, Especially the Good Kind. Biochemistry.

[38] Wei X., Viadero R.C., Buzby K.M. (2005). Recovery of Iron and Aluminum from Acid Mine Drainage by Selective Precipitation. Environ. Eng. Sci..

[39] Spark K., Johnson B., Wells J. (1990). Is Copper(II) Hydroxide an Intermediate in the Formation of the Hydroxide Chloride and Hydroxide Nitrate of Copper(II)?. Aust. J. Chem..

[40] Pham A.N., Rose A.L., Feitz A.J., Waite T.D. (2006). Kinetics of Fe(III) Precipitation in Aqueous Solutions at pH 6.0–9.5 and 25 °C. Geochim. Cosmochim. Acta.

[41] Kokhanovsky A.A., Zege E.P. (1997). Optical Properties of Aerosol Particles: A Review of Approximate Analytical Solutions. J. Aerosol Sci..

[42] De S., Das B. (2024). Dimerization of Fe(III) Ion in an Aqueous Medium: Mechanistic Modelling and Effects of Ligands. ChemPhysChem.

[43] Yu W., Xu L., Lei K., Gregory J. (2018). Effect of Crystallization of Settled Aluminum Hydroxide Precipitate on “Dissolved Al”. Water Res..

[44] Tang H., Xiao F., Wang D. (2015). Speciation, Stability, and Coagulation Mechanisms of Hydroxyl Aluminum Clusters Formed by PACl and Alum: A Critical Review. Adv. Colloid Interface Sci..

[45] McBryde W.A.E., Rohr J.L., Penciner J.S., Page J.A. (1970). Stability Constants of Three Iron(III) Salicylates. Can. J. Chem..

[46] Sovago I., Kiss T., Gergely A. (1993). Critical Survey of the Stability Constants of Complexes of Aliphatic Amino Acids (Technical Report). Pure Appl. Chem..

[47] Gorman J.E., Clydesdale F.M. (1984). Thermodynamic and Kinetic Stability Constants of Selected Carboxylic Acids and Iron. J. Food Sci..

[48] Santana-Casiano J.M., González-Dávila M., Millero F.J. (2004). The Oxidation of Fe(II) in NaCl–HCO_3_^−^ and Seawater Solutions in the Presence of Phthalate and Salicylate Ions: A Kinetic Model. Mar. Chem..

[49] Pham A.N., Waite T.D. (2008). Modeling the Kinetics of Fe(II) Oxidation in the Presence of Citrate and Salicylate in Aqueous Solutions at pH 6.0–8.0 and 25 °C. J. Phys. Chem. A.

[50] Johnson D.K., Stevenson M.J., Almadidy Z.A., Jenkins S.E., Wilcox D.E., Grossoehme N.E. (2015). Stabilization of Cu(I) for Binding and Calorimetric Measurements in Aqueous Solution. Dalton Trans..

[51] Kontoghiorghe C.N., Kontoghiorghes G.J. (2016). Efficacy and Safety of Iron-Chelation Therapy with Deferoxamine, Deferiprone, and Deferasirox for the Treatment of Iron-Loaded Patients with Non-Transfusion-Dependent Thalassemia Syndromes. Drug Des. Dev. Ther..

[52] Hathaway B.J., Billing D.E. (1970). The Electronic Properties and Stereochemistry of Mono-Nuclear Complexes of the Copper(II) Ion. Coord. Chem. Rev..

[53] Coimbra J.T.S., Brás N.F., Fernandes P.A., Rangel M., Ramos M.J. (2019). A Computational Study on the Redox Properties and Binding Affinities of Iron Complexes of Hydroxypyridinones. J. Mol. Model..

[54] Cohen S., Petoud S., Raymond K. (2001). Synthesis and Metal Binding Properties of Salicylate-, Catecholate-, and Hydroxypyridinonate-Functionalized Dendrimers. Chem. Eur. J..

[55] Chatterjee S.N., Agarwal S. (1988). Liposomes as Membrane Model for Study of Lipid Peroxidation. Free Radic. Biol. Med..

[56] Hwang H.-S. (2017). Advances in NMR Spectroscopy for Lipid Oxidation Assessment.

[57] Halliwell B., Chirico S., Crawford M.A., Bjerve K.S., Gey K.F. (1993). Lipid Peroxidation: Its Mechanism, Measurement, and Significance. Am. J. Clin. Nutr..

[58] Timoshnikov V.A., Kichigina L.A., Selyutina O.Y., Polyakov N.E., Kontoghiorghes G.J. (2021). Antioxidant Activity of Deferasirox and Its Metal Complexes in Model Systems of Oxidative Damage: Comparison with Deferiprone. Molecules.

[59] Timoshnikov V.A., Koshman V.E., Deriskiba A.A., Polyakov N.E., Kontoghiorghes G.J. (2026). The Effects of Salicyluric Acid, the Main Metabolite of Aspirin, on Lipid Peroxidation Induced by Iron and Copper Ions in a Lipid Membrane Model. Int. J. Mol. Sci..

[60] Mandal P., Kretzschmar J., Drobot B. (2022). Not Just a Background: pH Buffers Do Interact with Lanthanide Ions-a Europium(III) Case Study. J. Biol. Inorg. Chem..

[61] Verhagen J., Vliegenthart J.F.G., Boldingh J. (1978). Micelle and Acid-Soap Formation of Linoleic Acid and 13-L-Hydroperoxylinoleic Acid Being Substrates of Lipoxygenase-1. Chem. Phys. Lipids.

[62] Degrand L., Garcia R., Crouvisier Urion K., Guiga W. (2023). Dynamic Light Scattering for the Determination of Linoleic Acid Critical Micelle Concentration. Effect of pH, Ionic Strength, and Ethanol. J. Mol. Liq..

[63] Gabryel-Skrodzka M., Nowak M., Teubert A., Jastrzab R. (2022). Coordination Chemistry of Phosphate Groups in Systems Including Copper(II) Ions, Phosphoethanolamine and Pyrimidine Nucleotides. Int. J. Mol. Sci..

[64] Murugavel R., Choudhury A., Walawalkar M.G., Pothiraja R., Rao C.N.R. (2008). Metal Complexes of Organophosphate Esters and Open-Framework Metal Phosphates: Synthesis, Structure, Transformations, and Applications. Chem. Rev..

[65] Luangkamchorn S.T., Kitchotsakul A., Udomkanarat T., Saenrit T., Hono K., Kerdsomboon K., Auesukaree C. (2026). Phosphate Mitigates Iron and Zinc Toxicity in Saccharomyces Cerevisiae by Modulating Metal Bioavailability and Intracellular Defense Mechanisms. Ecotoxicol. Environ. Saf..

[66] Dawra R.K., Sharma O.P., Makkar H.P. (1989). Protein-Phosphate Complexes as an Inhibitor of Iron-Induced Lipid Peroxidation. Biochem. Int..

[67] Cheng I.F., Zhao C.P., Amolins A., Galazka M., Doneski L. (1996). A Hypothesis for Thein Vivo Antioxidant Action of Salicyclic Acid. Biometals.

[68] Baltazar M.T., Dinis-Oliveira R.J., Duarte J.A., Bastos M.L., Carvalho F. (2011). Antioxidant Properties and Associated Mechanisms of Salicylates. Curr. Med. Chem..

[69] Martell A.E., Seib P.A., Tolbert B.M. (1982). Ascorbic Acid: Chemistry, Metabolism, and Uses. Ascorbic Acid: Chemistry, Metabolism, and Uses.

[70] Kontoghiorghes G.J., Barr J., Baillod R.A. (1994). Studies of Aluminium Mobilization in Renal Dialysis Patients Using the Oral Chelator 1,2-Dimethyl-3-Hydroxypyrid-4-One. Arzneimittelforschung.

[71] Dalla Torre G., Mujika J.I., Formoso E., Matito E., Ramos M.J., Lopez X. (2018). Tuning the Affinity of Catechols and Salicylic Acids towards Al(Iii): Characterization of Al–Chelator Interactions. Dalton Trans..

[72] Ali Agha A.S.A., Khaleel S., Abdelaziz H.M.A., Alzweiri M., Qinna N.A., AlDabet G., El Khassawna T., Aburjai T. (2026). Toward a Molecular Framework of Systemic Multi-Organ Toxicity Induced by Chronic Aluminum Chloride Exposure. Molecules.

[73] Reinke C.M., Breitkreutz J., Leuenberger H. (2003). Aluminium in Over-the-Counter Drugs: Risks Outweigh Benefits?. Drug Saf..

[74] Sheppard L.N., Kontoghiorghes G.J. (1993). Competition between Deferiprone, Desferrioxamine and Other Chelators for Iron and the Effect of Other Metals. Arzneim.-Forsch..

